# teamNGS Balances Sensitivity for Viruses with Comprehensive Microbial Detection in Clinical Specimens

**DOI:** 10.3390/microorganisms13122854

**Published:** 2025-12-16

**Authors:** Julie Yamaguchi, Gregory S. Orf, Jenna Malinauskas, Maximillian Mata, Sonja L. Weiss, Kenn Forberg, Todd V. Meyer, Peter O. Wiebe, Illya Mowerman, Stanley J. Piotrowski, Daniel Glownia, Mary A. Rodgers, John Hackett, Yupin Suputtamongkol, Pakpoom Phoompoung, Selvamurthi Gomathi, Amrose Pradeep, Sunil S. Solomon, Nicholas Bbosa, Pontiano Kaleebu, Ambroise D. Ahouidi, Souleymane Mboup, Austin F. Sequeira, Arinobu Tojo, Gavin A. Cloherty, Michael G. Berg

**Affiliations:** 1 Infectious Disease Research, Abbott Laboratories, Abbott Park, IL 60064, USAgregory.orf@abbott.com (G.S.O.); maximillian.mata@abbott.com (M.M.); sonja.weiss@abbott.com (S.L.W.); todd.meyer@abbott.com (T.V.M.); peter.wiebe@abbott.com (P.O.W.); illya.mowerman@abbott.com (I.M.); daniel.glownia@abbott.com (D.G.); mary.rodgers@abbott.com (M.A.R.); gavin.cloherty@abbott.com (G.A.C.); 2 Abbott Pandemic Defense Coalition, Abbott Park, IL 60064, USA; yupin.sup@mahidol.ac.th (Y.S.); pakpoom.pho@mahidol.ac.th (P.P.); gomathi@yrgcare.org (S.G.); pradeep@yrgcare.org (A.P.); sss@jhmi.edu (S.S.S.); nicholas.bbosa@mrcuganda.org (N.B.); pontiano.kaleebu@mrcuganda.org (P.K.); ambroise.ahouidi@iressef.org (A.D.A.); souleymane.mboup@iressef.org (S.M.); 3 Faculty of Medicine, Siriraj Hospital Mahidol University, Bangkok 10700, Thailand; 4 YRG Care, Chennai 600031, India; 5 School of Medicine, Johns Hopkins University, Baltimore, MD 21218, USA; 6 Uganda Virus Research Institute, P.O. Box 49, Entebbe 256, Uganda; 7 MRC/UVRI & LSHTM Uganda Research Unit, P.O. Box 49, Entebbe 256, Uganda; 8 Institut de Recherche en Santé, de Surveillance Épidémiologique de Formations (IRESSEF), Dakar BP 7325, Senegal; 9 SlieaGen, LLC, Austin, TX 78728, USA; austin.sequeira@slieagen.com; 10 Institute of Medical Science, University of Tokyo, Tokyo 108-8639, Japan

**Keywords:** next-generation sequencing, virus discovery, target enrichment, metagenomics

## Abstract

Probe-based capture represents a highly sensitive and cost-effective approach for overcoming host background and enriching viruses in metagenomic NGS (mNGS) libraries. Using clinical specimens collected globally from patients with fever or respiratory illness, we generated mNGS libraries by random priming and Nextera XT tagmentation, followed by target enrichment (teNGS) with Comprehensive Viral Research Panel (CVRP) probes. Capture pool sizes and total reads were optimized, and libraries were initially sequenced separately. Using only 3–4% of reads required for standard mNGS, teNGS achieved increased sensitivity, 100–10,000× increases in depth, and >50% genome coverage for pathogens with titers ≥ 1000 cp/mL. Application to >2000 clinical specimens from various matrices and to contrived samples containing viruses absent from the CVRP probe set enabled detection of diverse viral families and established a minimum 65% nucleotide identity for hybridization, respectively. To save time and resources, teNGS and mNGS libraries were then combined into one sequencing run: *teamNGS*. In addition to streamlining the workflow, teamNGS also improved genome recovery. Coupling methods maintain the sensitivity and coverage for viruses achieved by enrichment alone while also ensuring comprehensive recovery of non-viral microbes. teamNGS has the potential to improve patient management and lower the rates of unnecessary testing and antibiotic use.

## 1. Introduction

Metagenomic next-generation sequencing (mNGS) has revolutionized infectious disease research and at times succeeded in providing clinically actionable information [1,2]. In theory, its ability to detect any known or novel microbe present in patient specimens could replace all molecular diagnostics [3]. Yet, a key obstacle in transitioning NGS from the bench to the bedside is both its selling point and its Achilles’ heel from an in vitro diagnostic standpoint: a limitless number of targets makes it impossible to validate the detection of all pathogens and establish performance metrics (e.g., quantification, limit of detection, efficiency, analytical sensitivity) typical of traditional molecular and serologic tests. To address this, the Galileo One^TM^ from ARC Bio takes an unbiased approach to sequence all microbes but limits its reporting to 1300+ microbial species. Labs will require skilled technicians, adequate space for sequencers, and ideally automation for library preparation. Beyond the need for dedicated software and pipeline development, subjective considerations around data interpretation abound, for example, accounting for run quality, establishing cut-offs for positive or negative results (and co-infections), and reconciling NGS results to clinical symptoms [4,5]. Numerous challenges unrelated to the science, including regulatory standards, certification, and insurance reimbursement, create additional barriers [6,7]. While CLIA approval has been obtained for narrow scope mNGS in certain teaching hospital settings, such as encephalitis [8,9] and respiratory disease [10], this is the exception, not the rule. There is currently no FDA-approved test that utilizes metagenomics. Despite these hurdles, and as evidenced by its widespread use in basic research, the promise of NGS has not been diminished.

With unbiased amplification of all nucleic acids in a sample, mNGS sensitivity for microbes is hampered by host genetic material typically present at much greater proportions. Numerous pre-extraction strategies to boost the signal-to-noise ratio, like nuclease treatment or filtration, have yielded incremental benefit [11,12]. Complementary post-extraction methods that seek to reduce human ribosomal RNA background or to boost detection with virus-specific reverse primers provide additional improvement [13,14]. Of the many approaches to improve NGS sensitivity, only positive selection with probe-based target enrichment (teNGS) has consistently provided substantial increases in genome coverage [15,16,17,18,19,20]. Capture of individual virus species or families is ideal for surveillance and comprehending intra-species diversity [21,22,23]. Previous iterations of probe-sets inclusive of all human/vertebrate viruses (VirCapSeq-VERT) were highly effective on contrived samples (and limited actual specimens) but initially proved prohibitively expensive due to the sheer number of probes (2 million) and scale of synthesis [15]. Metsky et al. devised the CATCH oligonucleotide design algorithm to reduce the number of probes required while preserving the ability to recover numerous, diverse sequences [24]. The target enrichment approach has since been applied to numerous clinical specimens and has recently been clinically validated for systemic and respiratory infections [25]. Congruent with panels detecting certain cancers or genetic diseases (e.g., cystic fibrosis), commercial kits scaling back capture probes to cover only a finite number of pathogens represents a middle ground approach. For example, the Karius Spectrum^®^ test contains >1000 pathogens that include bacteria, fungi, parasites, nematodes, and DNA viruses, but a key drawback is the exclusion of RNA viruses [26]. From Illumina, the Viral Surveillance Panel (VSP) is limited to 66 viruses with significant public health risk [27], and their Respiratory Pathogen ID/AMR Enrichment Panel (RPIP) focuses on respiratory infections from viruses, bacteria, fungi, and >2000 antimicrobial resistance alleles [28]. In essence, the number of organisms that can be targeted by probes in solution is seemingly limitless; it will simply depend on the application or disease state one chooses to interrogate [29,30].

Having played such a pivotal role in detecting and tracking the evolution of SARS-CoV-2 strains, the need to expand NGS and diagnostic capacity abroad to prevent outbreaks or mitigate their spread is clear [31,32]. Emerging and re-emerging RNA viruses such as coronaviruses, influenza viruses, Ebola virus, and dengue virus can mutate rapidly; thus, robust assays tolerant to genetic diversity and inclusive of all strains are needed to keep pace. Yet, as mNGS has revealed the wide expanse of viruses lurking in animal reservoirs and vectors, the next pandemic could be a virus never encountered before, resulting from a host jump for which no prior immunity exists [33]. Devising the means to screen individuals with undiagnosed illnesses in an automated, rapid, and cost-effective manner will be necessary to identify a new or re-emerging virus to have an impact on the trajectory of an outbreak [34]. In this study, we initially focused on implementing Twist Biosciences’ Comprehensive Viral Research Panel (CVRP) approach into our NGS workflow, highlighting its performance and suitability on thousands of clinical specimens. Our results concur with recent evaluations of CVRP showing similar limits of detection [20] and ≥10–100-fold boosts in on-target rates for viruses in mock samples [19], paraffin-embedded specimens [35], and mosquito vector homogenates [36]. However, existing commercial offerings and published methods cannot simultaneously achieve teNGS’s sensitivity for viruses and mNGS’s comprehensive detection of non-viral microbes. Recognizing the need to contain costs and devise a method that retains both properties, we combined target enrichment and metagenomics (teamNGS) to achieve these objectives on one sequencing run. Here, we are the first to describe the novel teamNGS approach and highlight its benefits.

## 2. Materials and Methods

Sourcing of samples. Patient specimens (e.g., plasma, serum, urine, whole blood, swabs in VTM) from individuals with confirmed infections or those presenting with acute febrile illness (AFI) or severe acute respiratory illness (SARI) originated from a variety of sources. Numerous samples were purchased from commercial vendors, including SlieaGen, LLC (Austin, TX, USA), Cerba Research (Paris, France), Boca Biolistics (Pompano Beach, FL, USA), Trina Bioreactives (Naenikon, Switzerland). Each vendor obtained the appropriate Institutional Review Board (IRB) protocol approval and patient consent for sample collection. Febrile patients (n = 7) having undergone hematopoietic stem cell transplant were sourced at the Institute of Medical Science (IMSUT) in Tokyo, Japan.

Nucleic acid extraction and preparation of mNGS libraries. Patient specimens were pre-treated with Benzonase (200 U/mL final, 37 °C for 3 h), and total nucleic acid (TNA) was extracted either on an *m*2000*sp* (Abbott Molecular, Des Plaines, IL, USA) as described [37] or on a KingFisher Apex using the MagMax Viral/Pathogen Nucleic Acid Isolation kit (ThermoFisher Scientific, Waltham, MA, USA) with a modified protocol that mimics that of the *m*2000*sp*. Each extraction of 48 or 96 contained 90 patient samples, four (1 per 24) positive controls (contrived virus cocktail each diluted to 3.0 log copies/mL), and two negative controls: healthy human plasma and a no-template control of phosphate-buffered saline (PBS). TNA was converted to double-stranded cDNA, and then to either Nextera XT or QuantaBio sparQ libraries using an epMotion liquid handler (Eppendorf North America, Enfield, CT, USA) as described [37]. Libraries were assessed, diluted, and loaded on Illumina instruments (MiSeq or NextSeq 1000) according to manufacturer recommendations and as described [37].

Preparation of teNGS and teamNGS libraries. The Comprehensive Viral Research Panel (CVRP; Twist Biosciences, South San Francisco, CA, USA), containing probes for >15,000 strains of vertebrate viruses, was used to generate teNGS libraries from mNGS libraries. Pooling, quantification, hybridization, and amplification steps were performed as previously reported [21,37]. For optimization experiments, mNGS libraries were pooled in batches of 8, 16, 24, or 32 before hybridization; otherwise, standard captures were in pools of 24 and libraries were sequenced in batches of 48 or 96 on MiSeq and NextSeq 1000 instruments. For teamNGS, we combined equimolar pools of target-enriched libraries with equimolar pools of their corresponding unenriched, metagenomic libraries into a single sequencing run. teNGS libraries (n = 24), already combined into a single tube following capture, were diluted to 2 nM. Individual mNGS libraries (n = 24) from the same samples were each diluted to 2 nM and equal volumes were collected into another pool. These equimolar pools of enriched (teNGS) and unenriched (mNGS) were then combined in teNGS:mNGS ratios of 1:9 or 2:8 *v*/*v* prior to the final dilution to 650 pM in RSB + Tween buffer, with an addition of ≤5% PhiX loading control.

Virus stocks and contrived specimen preparation and sequencing. Purified viruses with reported titers or TCID_50_ values were purchased from the ATCC (Encephalomyocarditis virus, EMCV, strain EMC, catalog number VR-129B; Zika virus, ZIKV, strain MR 766, catalog number VR-1838PQ) or large volume patient samples with viral loads determined by molecular assays were obtained (HIV-1 and SARS-CoV-2). Serial dilutions of EMCV, ZIKV, HIV-1, and SARS-CoV-2 were prepared and used as spike-ins to normal donor plasma to make contrived positive specimens. The EMCV stock had a concentration of 7.9 × 10^6^ TCID_50_/mL, and the ZIKV stock had a titer of 3.0 × 10^8^ cp/µL. HIV-1 was sourced from a high-titer (3.1 ×10^5^ cp/mL) plasma specimen from an individual in Cameroon, and SARS-CoV-2 was sourced from a high-titer (8.9 × 10^8^ cp/mL) swab-in-VTM specimen from a patient in the United States. Contrived positive specimens were prepared at 10,000, 5000, 1000, and 100 cp/mL from HIV-1, ZIKV, and SARS-CoV-2, while EMCV was prepared at 1000, 100, 10, and 1 TCID_50_/mL.

Virus and bacteria stocks and NGS positive controls preparation. Purified viruses and *Chlamydia* bacteria with reported titers or TCID_50_ values were purchased from the ATCC (Adeno-associated virus 2 strain H, VR-680; EMCV; Human adenovirus 4 strain RI-67, VR-1572; Human adenovirus 5, VR-1516; Rotavirus A strain Wa, VR-2018; BK polyomavirus, VR-837; Parechovirus A type 3 strain US/MO-KC/2012/006, VR-1886) or Exact Diagnostics (*Chlamydia*, ABB118 custom; VZV, ABB117 custom) and spiked into either patient plasma samples with known HIV-1 viral loads or normal human plasma at concentrations of log 3 cp/mL or log 4 cp/mL.

Serial dilutions for investigation of capture of viruses absent in CVRP probe set. Freeze-dried viruses absent or not well represented in the CVRP were purchased from the ATCC (Wanowrie strain G 700, Orthonairovirus, catalog number VR-445; Mount Elgon bat strain BP 846, Rhabdovirus, catalog number VR-627; Nodamura strain Mag115, Nodavirus, catalog number VR-679) and reconstituted in 1X PBS; one additional virus (Simian Hemorrhagic fever strain LVR 42-0/M6941, Arterivirus, catalog number VR-533) was received as infected TC fluid and cell lysate. Serial dilutions from 10^−1^ to 10^−5^ (*v*/*v*) in normal human plasma were prepared from each virus stock.

### Data Analysis

DiVir. NGS fastq files were processed for quality and removal of human sequences and remaining microbial reads were taxonomically classified using an in-house, proprietary metagenomics and virus discovery pipeline (DiVir 3.0) [38]. To enable designating a pathogen ‘positive’, reads were normalized (reads per million collected, RPM) and compared to levels present in other specimens on same run/cohort, as well as to the no-template control [9]. Those showing an *RPM-r* > 10 (RPM-*r*elative to negative control) or with representation from >3 independent genomic regions were evaluated by reference mapping in CLC Genomics Workbench v.23 (Qiagen Corp., Germantown, MD, USA). Iterative rounds of mapping performed at the defined stringencies were as previously described [23].

Plotting of NGS read mappings. After satisfactory reference mapping in CLC Genomics Workbench, the *QC for Read Mapping* module was used to produce various statistics such as reference coverage and coverage depth. Coverage plots were also exported as text files. Plotting of data was performed in either Microsoft Excel or the *R* (v.4.3.1) programming language (using primarily the *tidyverse* v2.0 set of libraries). Vector map data was obtained from the public domain Natural Earth service using the *rnaturalearth* v1.0 package for *R*.

Alignments of Orthonairoviruses and Rhabdoviruses. Reference genomes were retrieved from GenBank for the Orthonairovirus and Rhabdovirus species represented in the CVRP probe set. Multiple sequence alignments of comparable sequences from each group were performed using the L-INS-i algorithm of MAFFT v.7.487. Comparable sequences that produced >10% gaps across the alignments were removed from the analysis to prevent biasing downstream analyses. An *R* script was written utilizing the *Biostrings* v.2.6.81 and *tidyverse* v2.0 libraries to plot pairwise identity scores across each comparable Orthonairovirus genome against Wanowrie virus, or each comparable Rhabdovirus genome against Mt. Elgon bat virus. NGS coverage profiles of Wanowrie virus or Mt. Elgon bat virus were co-plotted against the pairwise identity profiles.

Statistical Analysis. For the Pearson’s product–moment correlation test, percent on-target values were standardized in two steps: first within each virus (scaling to the within-virus maximum), and then within each virus × level subgroup. “High” and “Low” levels corresponded to input concentrations of 5000 and 1000 copies/mL, respectively. An Aligned Rank Transform (ART) procedure was employed to compare across sequencing methods since the dependent variable, genome coverage, violated the assumptions required for parametric ANOVA. Datasets (n = 18) for mNGS, teamNGS (10%, P2), and teNGS were evaluated in three groupings: all together, viral only, and non-viral.

## 3. Results

### 3.1. CVRP Enables High Throughput and High Sensitivity Detection of Viruses

**Figure 1:**
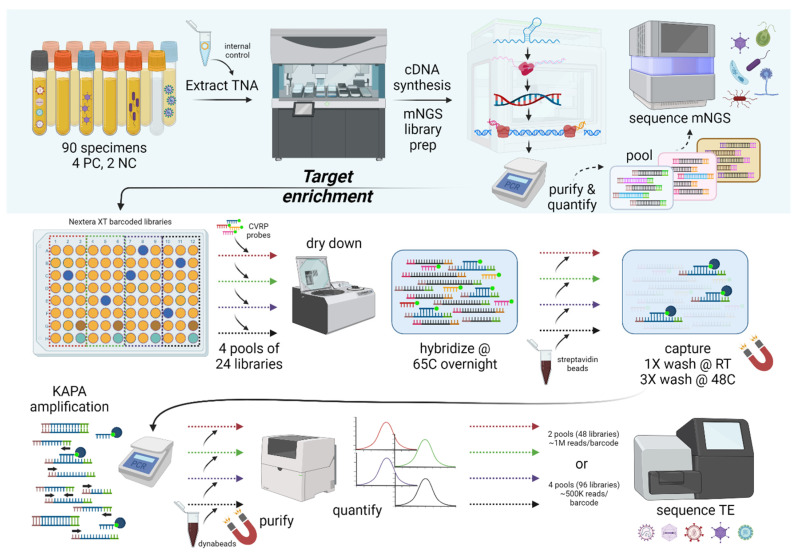
Automated mNGS and virus target enrichment workflow. Clinical specimens in batches of 96 are pre-treated with benzonase and spiked with an internal control before extraction of total nucleic acid on an Abbott *m*2000*sp* or KingFisher instrument. An epMotion liquid handler (in a pre-amplification room) is used to perform cDNA and second-strand synthesis steps. Following bead clean-up, Nextera XT ‘tagments’ double-stranded cDNA with barcoded i5/i7 adapters. Amplified libraries are purified by bead clean up (in a post-amplification room), quantified by Qubit and BioAnalyzer, diluted, and pooled for mNGS sequencing on a NextSeq. For virus target enrichment, equal volumes of mNGS libraries are pooled and dried down in a heated vacufuge in four batches of 24 (blue = virus positive; brown = NC; teal = PC). DNA pellets are reconstituted with CVRP probes and hybridized overnight at 65 °C, followed by the addition of magnetic streptavidin beads for 30 min. After a series of stringent washes, captured viral reads are amplified via PCR for 16 cycles using primers annealing to Illumina adapters. Library peaks are quantified, pools are diluted, and then all are combined onto MiSeq or NextSeq P1 runs.

Automation was leveraged to process clinical specimens and controls into NGS libraries in batches of 24, 48, or 96 (Figure 1). Extractions of total nucleic acid, performed on either the Abbott *m*2000 or the KingFisher Apex, are followed by reverse transcription, 2nd strand synthesis, and Nextera XT library preps on the Eppendorf epMotion liquid handler. Metagenomic libraries are sequenced to detect any microbe (i.e., virus, bacteria, fungi, parasite) and represent the starting material for the Comprehensive Viral Research Panel (CVRP) target enrichment protocol. Individually barcoded libraries are combined in pools of 24 (22 specimens + 1 positive control and 1 negative control), dried down by heated vacuum centrifuge, and hybridized to ssDNA probes complementary to >15,000 strains representing >3100 vertebrate virus species. Sample-derived viral reads captured by CVRP biotin-labeled probes are recovered with magnetic streptavidin Dynabeads while non-viral (host, bacterial, etc.) are removed by multiple wash steps. Bound viral sequences are then amplified with PCR using primers annealing to sites in Illumina adaptors. Two (n = 48 samples) or four (n = 96 samples) target-enriched pools are combined and sequenced on a MiSeq v2 flow cell or NextSeq P1 flow cell, respectively. Thus, viral reads are preferentially sequenced and the host background typically consuming the bulk of NGS datasets is removed. Sequencing performed on CVRP libraries will be referred to hereafter as “target-enriched NGS” (teNGS).

**Figure 2:**
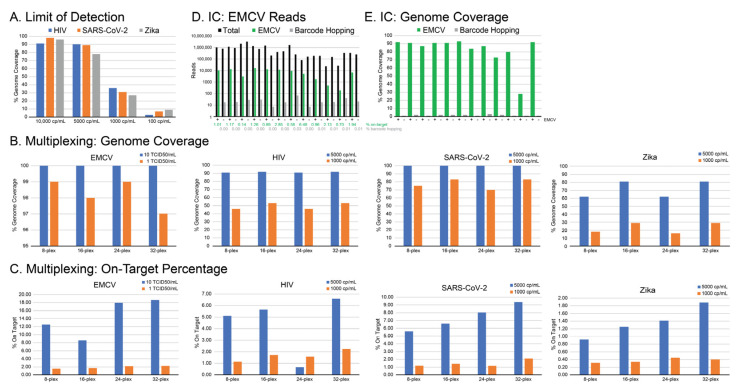
Performance of target enrichment on contrived specimens (**A**) Purified stocks of HIV, SARS-CoV-2, and Zika virus resuspended in healthy donor plasma were serially diluted and processed through the CVRP workflow to determine limits of detection in terms of genome coverage. (**B**) Dilutions of each virus stock at 5000 (blue) and 1000 cp/mL (orange) (and EMCV at 10 and 1 TCID_50_) were captured in pools 8, 16, 24, and 32 libraries to determine percent genome coverage and (**C**) on-target read percentages. (**D**) EMCV was spiked into the lysis buffer at 1 TCID_50_ to serve as an internal control for the entire procedure. Twelve libraries with and twelve without the EMCV IC were pooled, target enriched, and sequenced. Total reads (black) and EMCV reads in positive (green) and negative (grey) libraries and (**E**) EMCV genome coverage in positive libraries were measured.

The initial workflow defined in Figure 1 functioned as a starting point, but we set out to prove methodically which conditions were optimal and define performance on clinical specimens. Varying probe concentrations, hybridization temperatures, wash stringency, and PCR cycles either decreased efficiency (i.e., on-target rates) or provided minimal improvement, and thus we adhered to manufacturer recommendations. To mimic patient samples more closely, stocks of purified HIV-1, SARS-CoV-2, and ZIKV were serially diluted into healthy donor plasma to a titer of 10,000 (log 4), 5000 (log 3.7), 1000 (log 3), and 100 (log 2) cp/mL of each virus (Figure 2A). To address the limit of detection, individual mNGS libraries were captured in pools of 24 to obtain an estimated 1 million (MM) reads/per sample on a MiSeq. At log 4 cp/mL, 90–100% genome coverage was obtained for all three viruses. Coverage began to decrease between log 3.7 (80–90%) and log 3 (25–35%), consistent with similar virus teNGS methods [23]. At log 2 cp/mL, <10% of the genome was recovered, but reads from >3 independent regions of each virus were still detected.

For CVRP enrichment, increasing the level of multiplexing was the most important factor to address practicality and in turn was the most significant deviation from the manufacturer’s recommended protocol. Pooling only 8 libraries per capture fails to justify the added expense, time, and workload required of CVRP (Table 1). Initial experiments with pools of 24 libraries demonstrated feasibility, but we sought to prove whether smaller pools performed better or if it was possible to multiplex even more than 24. Libraries containing the same viruses (HIV-1, SARS-CoV-2, and ZIKV) diluted to 5000 cp/mL (log 3.7) and 1000 cp/mL (log 3) and EMCV at 10 and 1 TCID_50_/mL were interspersed among virus-negative and other unrelated virus-positive (low- and high-titer) libraries to achieve hybridization pools of 8, 16, 24, and 32 (Figure S1). Forty libraries, with each hybridization pool having different barcodes (^A^ or ^B^): (8^A^ + 32^B^ or 16^B^ + 24^A^) to distinguish identical dilutions, were sequenced together on two independent MiSeq runs (v2, 2 × 150 bp). The mass of each individual library was held constant during pooling (i.e., the final mass of the *library pool* was not the same). For each virus, genome coverage at both titers was not affected by the number of libraries multiplexed nor by the presence and titer of other samples in the pool (Figure 2B and Figure S1). Interestingly, as more libraries were multiplexed, the on-target percentage steadily increased, most noticeably at the higher titer (Figure 2C). A Pearson’s product–moment correlation test was performed which indicated a moderate positive correlation at the 5000 cp/mL titer (r = 0.51, 95% CI: 0.02–0.80, *p* = 0.044) that increased upon removal of an outlier (e.g., HIV at 24-plex) to r = 0.82, 95% CI: 0.52–0.94, *p* = 0.0002. At the 1000 cp/mL titer, a strong positive correlation was also observed (r = 0.77, 95% CI: 0.45–0.92, *p* < 0.001). Both titers demonstrate that higher multiplexing is associated with improved on-target performance. We attribute this to an overall increase in DNA concentration resulting from combining more libraries.

Confident the 24-plex pools were still an appropriate format (even though 32-plex was also acceptable), we incorporated EMCV as a ‘proof of concept’ internal control (IC) to measure recovery throughout the entire workflow. An equivalent of 1.0 TCID_50_/mL of EMCV was spiked into the lysis buffer onboard the *m*2000*sp* and therefore is present in every extracted sample. Total reads (black) following CVRP enrichment ranged from 100,000 to 1,000,000 per barcode and EMCV reads (green) ranged from 1000 to 10,000 per barcode (Figure 2D). Thus, EMCV consistently comprised ~1% of all reads and produced 90% genome coverage (Figure 2E). To rule out the effects of ‘index hopping’ [39], libraries lacking EMCV were sequenced on the same run and demonstrated that minimal cross-contamination (≤10 reads) was occurring (Figure 2E). Importantly, detection of other viruses in the sample was not affected by the presence of the IC (Supplemental Table S1) or vice versa. In capture sets of 24 libraries, a positive control consisting of a cocktail of five viruses (RNA and DNA), or four viruses (RNA and DNA) and one bacterium (*Chlamydia*), each diluted to log 3.0 cp/mL in normal human plasma (and a negative control), are processed alongside patient samples to gauge sensitivity and sample background (Supplemental Figure S2). We have successfully sequenced two or four CVRP pools of 24 (48 or 96 libraries) on a single MiSeq v2 or NextSeq P1 run at a full *reagent* cost of approximately 115 USD or 102 USD per patient specimen, respectively. In contrast, we routinely can only accommodate eight metagenomic libraries on a single MiSeq run (ensuring >2.5 MM paired-end reads per library to adequately detect low levels of virus), equating to a full *reagent* cost of approximately 228 USD per patient specimen.

### 3.2. Application of CVRP to Clinical Samples

**Figure 3:**
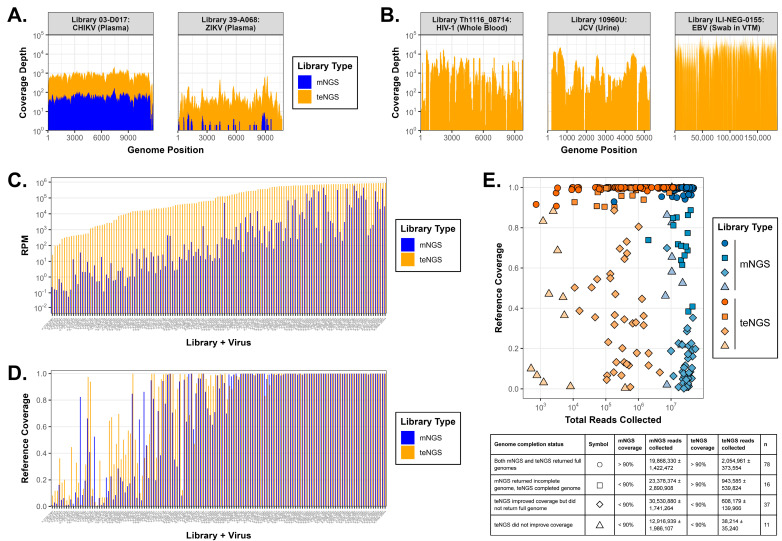
Performance of target enrichment on clinical specimens. (**A**) mNGS (blue) and teNGS (orange) coverage plots for Chikungunya (**left**) and Zika (**right**) virus infections. (**B**) teNGS coverage plots for HIV-1 in whole blood (**left**), JC Polyomavirus in urine (**center**), and EBV in nasal swabs (**right**). (**C**) AFI viral infections detected in Thailand patients were sequenced by both teNGS and mNGS, and results were plotted by reads per million (RPM) and (**D**) percent genome coverage. (**E**) Summary of sequencing method output relating total reads obtained to genome coverage. Symbols for categorization of genome completeness are denoted in the legend.

Metagenomic and target-enriched libraries were prepared from clinical specimens (plasma, serum) of suspected or confirmed cases sourced from several commercial vendors. teNGS via CVRP detected a wide range of viruses for which the results generally corroborated accompanying PCR and serology testing data (Supplemental Table S2). Discrepancies of note were samples with Rubella diagnoses that were found to actually contain Parvovirus B19, and ‘unknown hepatitis’ cases where low abundance Hepatitis B virus reads were now detected by CVRP. mNGS libraries sequenced in parallel either agreed with teNGS results or were not sensitive enough to detect the (presumably) low titer infections found by CVRP. Importantly, when a sample was negative (no viral reads) by teNGS it was also negative by mNGS. Infections with ZIKV (library 39-A068) and Chikungunya virus (CHIKV; library 03-D017) illustrate the substantial increases in coverage breadth and depth, respectively, with teNGS compared to mNGS (Figure 3A). These examples illustrate that (1) (+)ssRNA viruses are readily detected from plasma and serum (see also Dengue virus (DENV), West Nile virus, and Enterovirus A) and (2) the CVRP method (and Nextera XT preceding it) is agnostic to specimen matrix and virus genetic composition, also capturing small ssDNA (e.g., Parvovirus), reverse transcribing (e.g., HBV), segmented (−)ss/ds RNA (e.g., Rotavirus), and large dsDNA viruses (e.g., VZV) (Supplemental Table S2). Here it has been applied to whole blood (HIV-1), urine (JC Polyomavirus), and nasal swabs resuspended in VTM (Epstein–Barr virus), detecting common viruses from among these different genome types (Figure 3B). Viruses such as HIV-1 and DENV4 detected by teNGS in one specimen type (e.g., plasma) could be confirmed in another (e.g., whole blood), although we note the ‘choppy’, overamplified appearance in blood perhaps related to interfering substances [40] (Figure S3A). Finally, CVRP probes can be used with various library preparation kits, yielding comparable results for Illumina Nextera XT (tagmentation) or Quantabio sparQ (ligation), only requiring different adapter blocker oligos (Figure S3B,C). Taken together, CVRP is well suited for capturing a broad range of ‘known’ RNA and DNA viruses from various clinical sample types.

Samples from acute febrile illness patients (n = 900) sourced from Thailand, uncharacterized and without orthogonal testing, were also sequenced by both mNGS and teNGS to assess method metrics in a blinded study. Using the minimum criteria of recovering reads in at least three non-contiguous genome regions or ≥500 specific reads per million collected (RPM) and ≥10% coverage [20], a total of n = 141 viral infections, including DENV, CHIKV, and HAV, were detected by one or both methods. While in some cases viral loads or ELISAs were previously performed in Thailand, Abbott was blinded to these results. The on-target rate, represented by RPM, shows that teNGS consistently provides a 100–1000× boost in viral reads (median: 548×, range: 1.3–35,502×) compared to mNGS (Figure 3C). Thus, with teNGS, one might deduce that an equivalent number of viral reads and depth could be obtained with 100- to 1000-fold fewer allotted reads than with mNGS. Note that as the number of viral reads increased (>10% of total reads), this divide shrinks and the advantage of teNGS is diminished. Also, RPM values do not necessarily reflect coverage breadth, since teNGS read numbers could be inflated by overamplification and numerous reads mapping to identical regions (i.e., ‘pancaking’). Percent genome coverages were then plotted for each method (Figure 3D). Roughly two-thirds of the viruses (66%) obtained ≥90% coverage with teNGS. One population of libraries (left side; those with RPM values in teNGS < 50,000) gained a sizeable boost in reference coverage (median values, 21% to 45%, a 2-fold increase compared to mNGS). On the other extreme (right side), full genomes were obtained by both methods, suggesting no advantage in coverage for teNGS, but this ignores the total number of reads required in each case. These groupings likely trend with viral loads and suggest there are titer ranges where teNGS is well suited and others where it is unnecessary [20]. In summary, when mapped reads are normalized to total (RPM), teNGS vastly outperforms mNGS.

To determine the optimal number of total reads required for each method and estimate the number of samples to multiplex on a given platform, genome coverage was plotted versus the total absolute (not relative, RPM) number of reads collected (Figure 3E). In a sizeable subset of samples (55%), a full genome could be assembled with only 50,000–2,000,000 teNGS reads (median ~ 592,000), whereas 6–50 million mNGS reads (median = 13.3 million) were required (circles; see right side in Figure 3E). For what are likely high viral load samples (circles), it suggests that the actual ratio is closer to 30–100× fewer teNGS reads needed as compared to mNGS. Thus, 24–48 teNGS libraries can be comfortably run on a MiSeq that yields 40 million (20 MM paired-end) reads, whereas only 3–4 mNGS libraries can run for the needed throughput. Note the next subset (11%) denoted by squares, where complete genomes were again obtained by CVRP with as few as with 50,000–1,000,000 reads, but these same samples yielded incomplete genomes with ~20 million mNGS reads (squares). Hovering at 60–90% coverage suggests that full genomes for a variety of viruses would be achievable by sequencing deeper with mNGS, but this comes at a greater expense. This appears intrinsic to the sample and impossible to predict, but that sufficient coverage is observed after 10 million reads suggests this is a reasonable target for mNGS throughput. The next subset (26%) highlights CVRP’s improvement in coverage, albeit incomplete (diamonds; see left side of Figure 3E). The wide range in total teNGS reads (10,000–1,000,000) and coverage (10–80%) might indicate that more reads would result in greater coverage, but corresponding mNGS libraries remain at 0–30% coverage despite >20 million reads. This suggests teNGS coverage has reached a plateau for these samples and that any increase in mNGS coverage from sequencing deeper would yield diminishing returns. Finally, there was a minority of samples (8%) not improved by teNGS (triangles) which appear to represent a technical issue related to not obtaining enough reads, ranging in number from only 1000 to 10,000 teNGS reads, compared to the requisite 10 million mNGS reads. Taken together, most viruses were confidently identified (87% returning >20% coverage), if not completely covered by teNGS (66% returning >90% coverage), with only 10^5^–10^6^ reads, while approximately 10^7^ reads were needed to achieve the same with mNGS.

### 3.3. CVRP Utility for Virus Discovery

**Figure 4:**
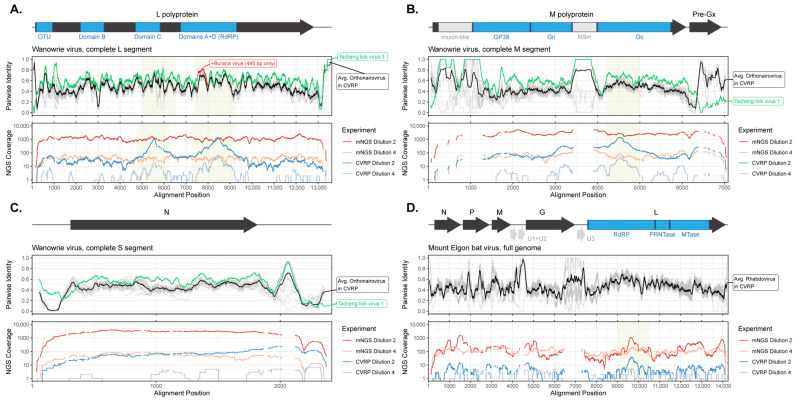
CVRP requires >65% nucleotide identity for enrichment of divergent viruses. Serial dilutions of Wanowrie virus and Mt. Elgon Bat virus were processed as described in Figure 1, and both metagenomic and CVRP target-enriched libraries were sequenced. Related orthonairovirus and rhabdovirus sequences, respectively, were retrieved from GenBank and their percent nucleotide identities to Wanowrie virus segments (**A**–**C**) and Mt. Elgon Bat virus (**D**) were determined using 120 nt sliding windows (**upper panels**; average in bold black line). Genome coverages (**lower panels**) for mNGS and CVRP were plotted for 1:100 (dilution 2; red) and 1:10,000 (dilution 4; blue) libraries. Yellow shading indicates regions of enrichment.

We next sought to establish the extent to which CVRP can recover novel, ‘unknown’ viruses. We hypothesized that probe cross-reactivity would impart a limited ability to capture genomic regions of high conservation present in divergent viruses. To test this, we selected viruses belonging to a variety of families for which specific probes were not present in CVRP. Ten-fold serial dilutions of stock concentrations for rhabdovirus (Mount Elgon bat), bunyavirus (Wanowrie), arterivirus (Simian Hemorrhagic Fever), and nodavirus (Nodamura) were then spiked into healthy plasma and captured by CVRP. In all cases, complete or near-complete genome coverage was obtained at the higher concentrations (10^−1^, 10^−2^ dilutions). At lower concentrations (10^−3^, 10^−4^ dilutions) we were surprised to still observe 50–100% coverage with RPM > 300 and frequently detected a few reads at the 10^−5^ dilution (Supplemental Table S3). To verify that this was attributable to probe-mediated capture and not to non-specific binding to streptavidin beads, each unenriched mNGS library at the 10^−2^ and 10^−4^ dilutions was sequenced to determine fold enrichment. Unlike the examples presented in Figure 3, these comparisons indicated enrichment was *not* occurring, since there was generally a several-fold *reduction* in CVRP RPM compared to mNGS. Nevertheless, we focused on the 10^−4^ dilution where partial genome coverage was obtained to understand whether our selected model viruses behaved in the same way (Figure 4, Supplemental Table S3).

For context, human sequences typically comprised ~80% of reads in mNGS libraries and decreased four-fold to ~22% in corresponding CVRP libraries (Supplemental Table S4). Somewhat unexpectedly, the percentage of bacterial reads held steady at ~1% in both libraries, whereas most divergent viral reads were efficiently removed by washing in the CVRP procedure. Nodamura virus read abundance for segments 1 and 2 decreased by 33- and 50-fold, respectively, and for Simian Hemorrhagic Fever virus they dropped by 25-fold, after CVRP (Supplemental Table S4). Probes for Nodamura virus and Arterivirus family members are completely absent in CVRP. By contrast, the percentage of reads for Mount Elgon Bat virus and Wanowrie virus only decreased by 12- and 4-to-8-fold, respectively (Supplemental Table S4). For Rhabdoviruses (Mount Elgon Bat), Ledantaviruses are absent but Lyssaviruses and Vesiculoviruses are present. For Bunyaviruses (Wanowrie), there is broad representation of each genus (e.g., Orthonairo-, Phlebo-, Hanta-, etc.) in CVRP, which suggested that probes against related viruses may be facilitating capture of the latter examples.

Reports of nucleotide identity thresholds required for hybridization are variable, depend on probe length, and often refer to cutoffs based on full genomes averaged together [15,41], rather than understanding the difference in probe capture across different regions of the genome. Here, comparing CVRP genome coverages and read depth for 10^−2^ and 10^−4^ dilutions data against percent identities for related viruses in the probe set, we empirically determined the regions and identity ranges that preferentially mediate capture. Individual pairwise identities (gray lines) between Wanowrie segments and Mount Elgon bat versus other orthonairoviruses or rhabdoviruses targeted by probes, respectively, were calculated from a multiple sequence alignment (MSA) using 120-nt sliding windows (Figure 4). For the Wanowrie L segment encoding RdRp, we observed prominent peaks for both CVRP dilutions within Domain C (positions 4750–6250) and Domains A and D (positions 7250–9250), which are absent in mNGS profiles and suggest enrichment. Twist Biosciences confirmed that probes for the closest relative (68% identity) from the Tamdy serogroup (Burana virus; KF801651.1 strain 760) were present but only covered Domain A + D. For the 60.3% genome coverage obtained at the 10^−4^ CVRP dilution, nearly half resided within these conserved intervals. For the M segment, CVRP coverage profiles at both dilutions once again resemble one another and contrast with mNGS. The peak in the N-terminus of Gc (positions 4200–5000) corresponds to the target location of Taching tick virus 1 probes that consistently share >70% identity with Wanowrie (Figure 4B). For the N gene in segment S, none of the close relatives share elongated stretches of elevated identity with Wanowrie, and thus specific enrichment did not occur. (Figure 4C). In the case of Mt. Elgon bat virus, low coverage depths for CVRP at each dilution mirror that of their corresponding mNGS libraries, except for the RdRp region from positions 9000–10,000, where the average nucleotide identity approaches 55% (Figure 4D). Focusing on intervals of ≥1000 nucleotides in these two examples and assessing the percent identity of the closest reference, we determined that >65% identity across such a contiguous region is required for hybridization (Supplemental Table S5).

From a febrile patient sample (C15-17230099) evaluated in this study for which no known cause of infection was detected, we identified a novel, highly divergent bunyavirus most closely related to an Orthodiscovirus recently found in the fungus *Penicillium roseopurpureum* [42]. The RdRp (L) and nucleocapsid (S) segments were readily assembled from mNGS reads, which notably also detected the presence of numerous fungal plant pathogens (e.g., *Didymella pinodes*, *Coniothyrium glycines*, *Fusarium* spp.) causing crop blight, suggesting this could be a dietary contaminant. Consistent with other reports [43], we were unable to detect a third 1.7 kb M segment encoding the non-structural protein NSs. CVRP reads were orders of magnitude lower (Figure S4) than mNGS, a phenomenon we have also observed with a novel cyclovirus found in Cameroon [44]. With amino acid identities (not nucleotide) to known discoviruses being only 62.7–75.6%, CVRP probes for vertebrate viruses with sufficient nucleotide identity are not expected. Therefore, the limited coverage obtained for divergent viruses is often due to non-specific binding, which one could argue is a positive attribute of the method. Predictions for the likelihood of capture based solely on its ‘overall’ genomic percent identity to other viruses in the probe set are imperfect, since what is actually required are extended regions of conservation (>65% identity) within the virus to mediate capture. Thus, our data suggests that teNGS has limited utility when used as the sole vehicle for virus discovery.

### 3.4. CVRP Reveals a Wide Variety of Viruses Present in Undiagnosed Illnesses

**Figure 5:**
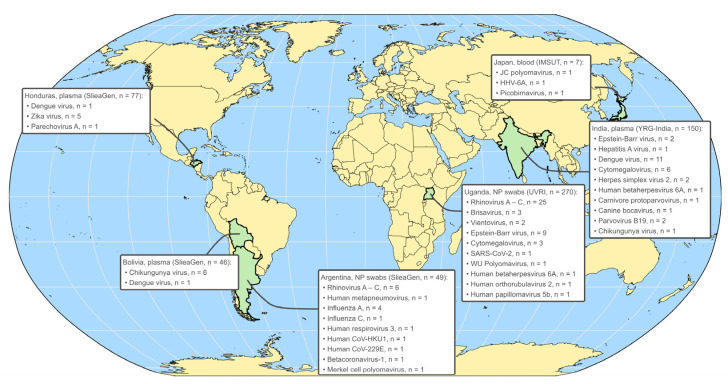
CVRP detects a wide range of viruses among cases of unexplained illness. Target enrichment was applied to 599 specimens sourced from around the world exhibiting symptoms of respiratory (Argentina, Uganda) or acute febrile (Honduras, Bolivia, India, Japan) illness. Viral species and number of positives detected in each country are shown in boxes.

We have applied mNGS with and without target enrichment to thousands of clinical specimens from several study cohorts exploring illnesses of unknown origin (Figure 5). For presumed arbovirus infections from Bolivia and Honduras in 2016, plasma was collected from patients who presented with symptoms of fever, arthralgia, headache, nausea, retroorbital pain, etc. In Honduras (n = 77), we detected DENV (n = 1), ZIKV (n = 5), and parechovirus (n = 1), while in Bolivia (n = 46) we detected CHIKV (n = 6) and DENV (n = 1). Acute upper airway infections from Argentina (n = 49) pre-screened for SARS-CoV-2 revealed a wide variety of respiratory viruses, including rhinoviruses (A, B, C), metapneumovirus, influenza viruses (A, C), respirovirus-3, and seasonal coronaviruses (HKU1, 229E), for which CVRP yielded full genome sequences. A larger screen of individuals (n = 270) from Uganda with influenza-like illness and severe acute respiratory illness (ILI/SARI) ascribed a viral cause to n = 44 (16.3%) samples. Rhinoviruses (A, B, C) explained most cases, with EBV, CMV, human rubulavirus-2, and HHV-6b also being detected. Members of the recently described *Redondoviridae* (brisavirus and vientovirus), a family of circular DNA viruses frequently found in respiratory tracts, were also detected [45].

Turning to Asia, in a 2014 Japanese cohort of hematopoietic stem cell transplant patients experiencing episodes of febrile illness, we identified viruses previously reported to reactivate following this procedure [46,47,48,49]. Partial genome coverages were obtained for JC polyomavirus-2 (JC-PyV2), human herpesvirus 6B (HHV-6B), and human picobirnavirus (PBV) in three different patients. JC-PyV2 was detected by CVRP and confirmed by mNGS but not by PCR; HHV-6B was detected by mNGS and confirmed by PCR but not by CVRP; PBV was only detected by mNGS (Figure S5A). Finally, patients (n = 150) visiting emergency rooms in seven hospitals in India during 2022 presenting with acute fever and a variety of other symptoms were sequenced by CVRP. DENV was prevalent (n = 11), but herpesviruses (HSV-2, HHV-4, HHV-5, HV6A), CHIKV, hepatitis A, and parvovirus B19 infections were also observed (Figure 5). Detection of a canine protoparvovirus/feline panleukopenia parvovirus infection was rather unexpected. Nevertheless, we obtained 93% genome coverage at 25× depth, and the consensus sequence aligned by BLAST (https://blast.ncbi.nlm.nih.gov/Blast.cgi accessed on 6 June 2024) to strains reported from Thailand (MN127779.1) and India (MH559110.1) (Figure S5D). This individual had a fever (>1 week), chills, headache, nausea, and vomiting and a history of exposure to chickens but not to dogs or cats. A related but distinct canine bocavirus was detected 2 days later in a male roughly the same age in the same town (Thane), and 33% coverage was obtained (Figure S5B). This individual also exhibited fever and chills, as well as encephalitis and a rash. Probes for both viruses are included in CVRP and none of the remaining samples in the cohort were positive. Including positive and negative samples sequenced either individually or by both mNGS and teNGS, including those reported in Table S2, the Thailand AFI samples in Figure 3, and the cohorts described in Figure 5, these methods were applied to >2000 clinical specimens for this study, resulting in 125 new full viral genome submissions to GenBank.

### 3.5. TeamNGS Maximizes Virus Sensitivity and Retains Non-Viral Detection

**Figure 6:**
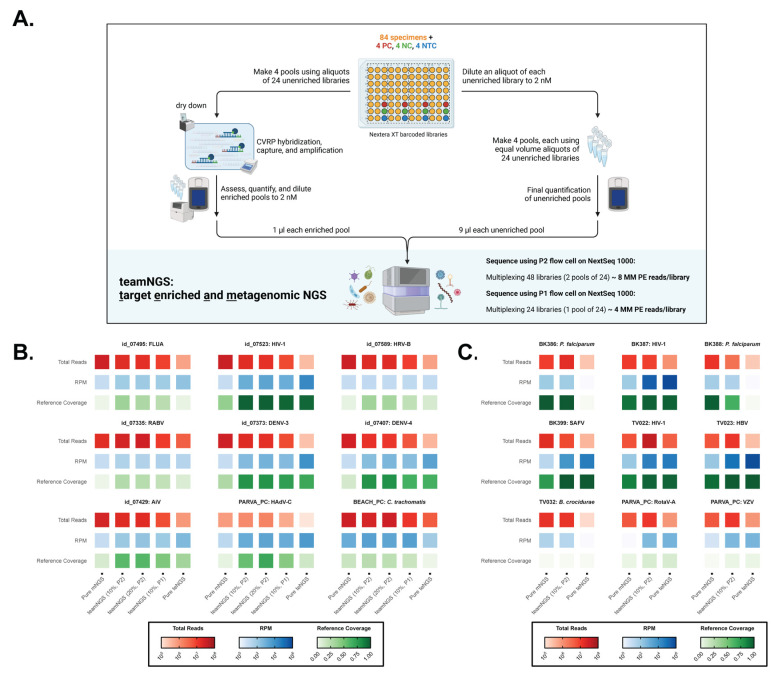
teamNGS improves coverage for viral genomes and maintains non-viral detection. (**A**) Workflow describing the combination of target-enriched and mNGS libraries into one sequencing run to obtain the desired throughput according to sample multiplexing. (**B**) Nine infections from Thailand were evaluated for total reads (red), RPM (blue), and reference coverage (green) in five separate sequencing experiments: 100% mNGS, teamNGS with 10% CVRP on a P2, teamNGS with 20% CVRP on a P2, teamNGS with 10% CVRP on a P1, and 100% CVRP. (**C**) Nine infections from Senegal were similarly compared in three experiments: 100% mNGS, teamNGS with 10% CVRP on a P2, and 100% CVRP.

While CVRP and mNGS complement each other (and having each dataset increases the confidence in an infection determination), the time and expense to sequence and analyze both sets of libraries independently becomes prohibitive at scale (Table 1). We therefore explored the efficacy of pooling the target-enriched and metagenomic libraries together on one run: *teamNGS* (Figure 6A). Reasoning that separately ~10 million reads are required for mNGS and ~1 million reads for CVRP, equimolar proportions of 90%/10% and 80%/20% (*v*/*v*), respectively, were tested initially. Resequencing Thailand libraries previously evaluated by both approaches, we also varied levels of multiplexing via NextSeq flow cell selection (P1 vs. P2) to adjust the available output of total reads (Figure 6B). Representative examples show that as total reads decrease, from pure mNGS to pure teNGS (CVRP), viral RPMs trend in the opposite direction, as expected. Interestingly, genome coverages from teamNGS libraries were often improved compared to teNGS alone (e.g., for Aichi virus, Adenovirus C, and Influenza A virus), suggesting an additive effect where distinct subsets of viral reads are drawn from both libraries (Figure S3C). Note for *Chlamydia trachomatis* that bacterial coverage is not negatively impacted when spiking in 10–20% CVRP reads. The differences observed for 90/10 versus 80/20 ratios were trivial, but coverage for several viruses did increase when graduating from a P1 (4 MM paired-end reads allotted per index) to a P2 (8 MM paired-end reads estimated per index) flow cell, indicating that allotting more reads was beneficial. To repeat these results, a 90/10 teamNGS run on a P2 flow cell was performed on leftover libraries originating from a recently characterized AFI cohort from Senegal [37]. Once again, teamNGS matched or improved the viral on-target rates and coverages achieved with teNGS alone (Figure 6C). Similarly, sensitivity for bacteria (*Borrelia crocidurae*) and parasites (*Plasmodium falciparum*) with mNGS alone was also preserved. To determine whether the increase in genome coverage with teamNGS was statistically significant, an Aligned Rank Transform test was performed (Supplemental Table S6). Across all pathogens, teamNGS (10%, P2) consistently produced higher aligned-rank genome coverage than both pure mNGS (*p* = 0.0004) and pure teNGS (*p* = 0.0150), whereas the individual methods did not differ significantly from each other (*p* = 0.2025). When only viral infections were considered, teamNGS was once again superior to teNGS (*p* = 0.0201) and mNGS (*p* = 0.0001). No significant differences in coverage were observed for non-viral pathogens, indicating teamNGS does not sacrifice sensitivity for them. Therefore, this simple additional step of merging libraries (Figure 6A) reduces both hands-on and analysis times and cuts sequencing reagent expenses in half (e.g., 1 run instead of 2), yielding data of the same or better quality.

## 4. Discussion

Unlike metagenomic NGS which lists a myriad of microbes and whose results are open to wide interpretation, CVRP target capture often succeeds in delivering a simple binary ‘yes/no’ answer and identifies which of >3100 vertebrate viral species is present in a sample. Here, we defined an optimized workflow that enables production of target-enriched libraries from mNGS libraries using the CVRP kit, both of which can be sequenced to provide complementary results and a holistic view of patient status. Larger-than-recommended multiplexing (e.g., 24–32 vs. 8 samples) for CVRP capture not only yielded highly sensitive results but surprisingly enhanced on-target rates in our hands, rather than diminishing it (Figure 2). A further advantage of distributing sequencing throughput is that with fewer total reads per barcode, it lowers the computational cost and complexity of taxonomic assignment. Given that, at times, the percentage of viral reads approached 100% in teNGS libraries, sequence depths greater than 500 M–1 MM reads are not only unnecessary, but in fact can be detrimental since they increase the likelihood of index hopping. While we observed this effect at a rate of only 1–10 RPM (Figure 2E), it is known to be higher (e.g., 1/10,000 reads, or 100 RPM) and is more noticeable and problematic in capture experiments when human background is removed [50]. With uncharacterized libraries amplified post capture, it is impossible to avoid the occasional ‘hot’ sample(s) that dominates a sequencing run. However, we demonstrated that while these can siphon off reads for other barcodes within a capture, it does not appreciably alter detection of other infections in the pool or sequencing run (Figure 2). Nevertheless, when this information is available, we recommend grouping samples by viral load and avoiding enriching high-titer samples. The CVRP method proved effective for >2000 clinical specimens derived from a variety of matrices (Figure 3 and Figure 5) and its proficient adoption by other groups (Figure 5) demonstrates that the method is robust.

The teamNGS concept was born out of the desire to reduce costs and streamline our workflow while maintaining the best attributes of each approach (Figure 6). Despite mNGS and teNGS both being well-established methods, to our knowledge, research combining them and illustrating the benefits of this approach directly on clinical specimens has not been published. We highlighted several examples where viral reads were low or non-existent in mNGS libraries and for which target enrichment dramatically improved sensitivity (Figure 3E and Figure 6B,C), both in terms of RPM and coverage. Now with teamNGS, this advantage does not require a separate run or need to come at the expense of diminished detection for bacteria and parasites. The benefits also extend to divergent viruses and those for which probes are absent, achieving increased genome coverage through complementation, wherein overlapping and distinct sets of reads are recovered (Figure 6B,C and Figure S3B). Recommendations for the number of mNGS reads per index has ranged from 5 to 10 million, largely driven by a lack of sensitivity for viruses [9,51]. Indeed, a sizeable fraction of samples (25%) with presumed low viral loads achieved less than 30% coverage by mNGS, even with 20+ MM paired-end reads (Figure 3E); sequencing deeper and trading off with lower multiplexing is unlikely to substantially improve this. Here we show that ~8 MM paired-end reads, of which 10% originate from CVRP captures, maintain comparable sensitivity for viral and non-viral species. This equates to 48 samples multiplexed on a NextSeq P2 flow cell, or 2.5 Gb data acquired per library. Assuming automation, CVRP captures of 24, and varying the degrees of multiplexing for sequencing, Table 1 describes expenditures in USD in terms of days, library prep reagents, sequencing reagents, server time, and data processing. First, the price per sample drops substantially when scaling up from 8 to 96 samples (474.98 USD vs. 201.56 USD) and the added per sample cost of target enrichment is not prohibitive (42.34 USD). Compared to the combined cost of sequencing individually by both methods, teamNGS delivers the same data with a savings of 1586 USD (30 USD/sample) to 1922 USD (22 USD/sample) per experiment, depending on whether 48 or 96 samples are processed. The biggest savings are realized in terms of time, cutting the workflow and analysis by 4.5–7 days per experiment. Over the course of a year (261 working days), this will allow one scientist to process 325 more samples (1252 vs. 928) or save the lab over 18,500 USD to perform the same number.

Target capture demonstrated that viruses were the putative etiologies for about 30% of unresolved cases of illness in our globally sourced patient cohorts (Figure 3 and Figure 5). In acute febrile and respiratory illness cohorts several anticipated pathogens were detected such as Zika and Chikungunya viruses or rhinovirus and metapneumovirus, respectively. In many resource-limited locales, screening consists of only Dengue/malaria or COVID-19/flu rapid tests, and individuals testing negative are unlikely to receive follow-up testing or a definitive diagnosis. While syndromic panels (e.g., those on the BioFire platform) probe for more pathogens, multiple positive results (i.e., possible co-infections) can confound interpretation. Most will go on to resolve their infection, but the ability to at least discriminate viral versus bacterial causes could lessen the over-use of antibiotics that is contributing to widespread resistance [52]. Also, though most illnesses have overlapping, indistinguishable flu-like or AFI symptoms, determining specifically which virus is infecting a patient could influence patient management and avoid long-term sequelae. For example, knowledge of the infecting Dengue virus serotype would improve risk assessment for Dengue hemorrhagic fever upon re-infection with a different serotype, as would knowing if someone has mononucleosis (EBV) and whether their symptoms (e.g., severe fatigue) may persist for weeks. Unexplained fevers resulting from the reactivation of dormant viruses following stem cell transplant (e.g., HPyV-2, HHV-6B) is a recurring theme and one we also observed [47,49] in samples from Japan. Unexpectedly, we identified feline and canine parvoviruses in people from India, a result that deserves follow-up and scrutiny, but likely would have been missed without target enrichment [53,54]. At present, research to understand the incidence or (re-)emergence of viruses, the detection of adventitious viruses in biological preparations (e.g., vaccines or recombinant proteins), or generating full genomes for phylogenetic studies, are realistic use-cases for the implementation of target enrichment technology such as CVRP.

The CVRP kit’s utility for discovering new viruses is limited, however. Others have reported that probe-based target enrichment strategies can capture genomes with identity of 80% [41], 60% [15], or even 42% [18]. However, our experiments suggest the tolerance for mismatches is lower and that non-specific binding to probes play a significant role in detection, depending on the abundance of the virus (Figure 4 and Figure S4). Diluting samples to evaluate non-saturating conditions and analyzing nucleotide alignments with related species, we demonstrated that only conserved sub-genomic regions mediate selective hybridization (Figure 4). Non-structural or enzymatic genes such as RdRp with >65–75% identity to nearest neighbors in the probe set were enriched, a cut-off value for divergence in line with Metsky et al. but more relaxed than reported by Twist Biosciences’ internal research using the Influenza virus HA gene (80%) [24,55] and others addressing this question with viruses not represented in the probe set (90%) [15]. Our value refers to a specific region of sufficient length, not the average overall genome identity. Thus, in line with others, we observed obtaining full length sequences of novel viral species or genera is not a reasonable expectation, but capturing evolutionarily conserved portions of the viral genome that can lead to a discovery is [15]. The practical implication of this 65% identity threshold for probe design is that using an alignment of related viruses to generate a consensus sequence is preferable to tiling individual genomes [23]. Here again, the case for teamNGS is self-evident, wherein regions that elude CVRP capture may be recovered by mNGS. Other limitations of our study include the selection of EMCV as the internal control (IC). This was intended as a proof-of-principle and a matter of convenience, but a more appropriate choice would be a non-vertebrate (e.g., insect or bacterial) virus [9]. In addition to demonstrating equivalent recovery throughout the process, the IC could also be used to convert teNGS into a quantitative assay in conjunction with calibrated positive control viruses. Our data is also focused on short-read technology and only assesses one probe set whereas others have explored target enrichment with different platforms [19] and capture panels [20]. Finally, our clinical samples generally lacked a definitive diagnosis and therefore we did not have prior diagnostics results (e.g., RT-PCR, serology, culture) to compare against, precluding a robust assessment of NGS performance in terms of sensitivity, specificity, and positive and negative predictive values. However, relative to mNGS or teNGS results, teamNGS detection metrics on the same samples were equal to or better than when performed individually (Figure 6B,C).

Targeted sequencing of SARS-CoV-2 played a pivotal role in tracking variants and the evolution of the virus around the globe. Whether generated via target enrichment or amplicon-based methods (e.g., ARTIC), this experience spoke to the need to harmonize methods to produce quality genomes. In the post-COVID era, as the pressures leading to, or exacerbating, outbreaks (e.g., global travel, expanding range of insect vectors, etc.) have not gone away, the scientific community needs to be better prepared [56]. International networks (e.g., Abbott Pandemic Defense Coalition, APDC; Centers for Research in Emerging Infectious Diseases, CREID Network; Global Alliance for Preventing Pandemics, GAPP) have been formed to equip more labs not only with the ability to sequence, but to also spot concerning infections with pandemic potential. Just as metagenomic NGS is likened to ‘finding the needle in the haystack’, so too is spotting the index case(s). Adherence to clinical case definitions, pre-screening to exclude the ‘usual suspects’ (e.g., Dengue, malaria, influenza), collection of metadata, and recognizing concerning epidemiologic trends are imperative pre-analytical measures. Finding these individuals will give the scientific community a head start on developing diagnostics and learning how to treat or prevent spread. Indeed, integrating case finding with sequencing into the existing public health systems has enabled early detection [57], with mortuary surveillance in Uganda spotting outbreaks of Echovirus 7 [38] and anthrax [58], and outpatient fever clinics in Colombia detecting Oropouche [34] and Mayaro [59] viruses. teamNGS should figure prominently among the tools geared towards these efforts and is well positioned to bridge research to the bedside and impact clinical decision making [51,60]. Despite examples of its clinical utility, metagenomic NGS is largely implemented long after one has already recuperated or expired and thus is still some distance from becoming standard of care. Even focusing NGS on a particular syndrome (e.g., encephalitis) or limiting its application to a particular specimen matrix (e.g., cerebrospinal fluid) will require surmounting significant logistical and financial hurdles involving regulatory and reimbursement paths. Nevertheless, the power and comprehensiveness of this technology is unquestionable, and given time, its transition to the clinic should prove possible.

## 5. Conclusions

Metagenomic NGS (mNGS), permitting detection of theoretically any organism with a nucleic acid genome, and target-enriched NGS (teNGS), selectively amplifying pathogens through probe-based hybridization, are both established molecular methods for analyzing patient specimens. However, the considerable time and expense to perform both these complementary approaches becomes prohibitive when scaling up and attaining the necessary read depth. *teamNGS* (target-enriched and metagenomic NGS) combines these two workflows into one sequencing run and data analysis step, with the results proving to be more than the sum of its parts. We detected all microbes in one experiment and achieved better metrics for on-target rates and viral genome coverage. The robust method has been applied to thousands of clinical specimens, to different sample types and library preparations, and implemented at different research sites, demonstrating its potential for widespread adoption.

## 6. Patents

A provisional patent has been filed on the teamNGS method.

## Figures and Tables

**Table 1:** Cost breakdown for CVRP Cost comparison of teNGS and mNGS separately versus teamNGS. In teNGS, metagenomic libraries must first be constructed before target enrichment can occur, thus one may reflex back to leftover aliquots of the metagenomic libraries for a second sequencing experiment. In teamNGS, the target-enriched libraries and their corresponding metagenomic counterparts are multiplexed together in a single sequencing experiment. Bioinformatics (BFX) costs are associated with on-demand usage of AWS computing resources for running sequencing analysis pipelines. Recommended sequencing kits and throughput are indicated, as are the estimated time needed to complete each step.

	Multiplexing 8 Libraries		Multiplexing 48 Libraries		Multiplexing 96 Libraries	
teNGS using CVRP	Total cost	Cost (and PE reads) per sample	Total cost	Cost (and PE reads) per sample	Total cost	Cost (and PE reads) per sample
Nucleic acid extraction	$14.98	$1.87	$89.76	$1.87	$179.52	$1.87
Metagenomic cDNA and library preparation	$518.16	$64.77	$3108.96	$64.77	$6217.92	$64.77
Laboratory time, metagenomic preparation	3 days		3 days		3 days	
Target enrichment kit (CVRP)	$535.68	$66.96	$1071.36	$22.32	$2142.72	$22.32
Laboratory time, target enrichment	2 days		2 days		2 days	
MiSeq, v3 (20 MM)	$1289.00	$161.12 (2.5 MM PE reads, teNGS)	inadvisable		inadvisable	
NextSeq 1000, P1 (100 MM)	$1250.00	$156.25 (12.5 MM PE reads, mNGS)	$1250.00	$26.04 (2.1 MM PE reads, teNGS)	$1250.00	$13.02 (1.0 MM PE reads, teNGS)
NextSeq 1000, P2 (400 MM)	inadvisable		$3628.00	$75.58 (8.3 MM PE reads, mNGS)	$7256.00	$75.58 (8.3 MM PE reads, mNGS) ^a^
Sequencing time, teNGS	1 day		1 day		1 day	
BFX analysis time, teNGS	0.1 days		1 day		2 days	
BFX analysis cost, teNGS	$56.00	$7.00	$336.00	$7.00	$672.00	$7.00
Human analysis time, teNGS	0.5 days		2 days		4 days	
Sequencing time, mNGS	1 day		2 days		2–4 days	
BFX analysis time, mNGS	0.1 days		0.5 days		1 day	
BFX analysis cost, mNGS	$136.00	$17.00	$816.00	$17.00	$1632.00	$17.00
Human analysis time, mNGS	1 day		5 days		10 days	
Total cost for preferred workflow	$3799.82	$474.98	$10,300.08	$214.59	$19,350.16	$201.56
Total time for perferred workflow	~9 days		~16.5 days		~27 days	
Added cost of using target enrichment	$1880.68	$235.09	$2657.36	$55.36	$4064.72	$42.34
Added time of using target enrichment	~3.5 days		~6 days		~9 days	
						
	**Multiplexing 8 libraries**		**Multiplexing 48 libraries**		**Multiplexing 96 libraries**	
teamNGS	Total cost	Cost (and PE reads) per sample	Total cost	Cost (and PE reads) per sample	Total cost	Cost (and PE reads) per sample
Nucleic acid extraction	$14.98	$1.87	$89.76	$1.87	$179.52	$1.87
Metagenomic cDNA and library preparation	$518.16	$64.77	$3108.96	$64.77	$6217.92	$64.77
Laboratory time, metagenomic preparation	3 days		3 days		3 days	
Target enrichment kit (CVRP)	$535.68	$66.96	$1071.36	$22.32	$2142.72	$22.32
Laboratory time, target enrichment	2 days		2 days		2 days	
NextSeq 1000, P1 (100 MM)	$1250.00	$156.25 (12.5 MM PE reads, mNGS)	inadvisable		inadvisable	
NextSeq 1000, P2 (400 MM)	inadvisable		$3628.00	$75.58 (8.3 MM PE reads, mNGS)	$7256.00	$75.58 (8.3 MM PE reads, mNGS) ^a^
Sequencing time, teamNGS	1 day		2 days		2–4 days	
BFX analysis time, teamNGS ^b^	0.1 days		0.5 days		1 day	
BFX analysis cost, teamNGS	$136.00	$17.00	$816.00	$17.00	$1632.00	$17.00
Human analysis time, teamNGS	1 day		5 days		10 days	
Total cost for preferred workflow	$2454.82	$306.85	$8714.08	$181.54	$17,428.16	$181.54
Total time for perferred workflow	~7 days		~12 days		~20 days	
						
All in optimal teNGS + mNGS cost	$3799.82		$10,300.08		$19,350.16	
All in optimal teNGS + mNGS time	~9 days		~16.5 days		~27 days	
All in optimal teamNGS cost	$2097.70		$8714.08		$17,428.16	
All in optimal teamNGS time	~7 days		~12 days		~20 days	

^a^ to achieve > 8 MM PE reads, two sequencing experiments with 48 libraries each must be run. ^b^ assuming that FASTQ from 10 libraries can be analyzed by the BFX pipeline simultaneously. PE = paired-end.

## Data Availability

The authors confirm that all supporting data, code and protocols are contained within the article or in the supplemental information. For those samples with >90% viral genome coverage, human-depleted raw sequencing data has been deposited in SRA. BioProject PRJNA1328708 contains n = 106 BioSamples (SAMN51281085-SAMN51281190) with n = 117 corresponding SRA entries (SRR35424513-SRR35424626). GenBank accession numbers (PX404475-PX404574, PX354160, and PX363308-PX363324) for these genomes are linked to the BioSamples in Supplementary Table S6.

## Supplementary Materials

The following supporting information can be downloaded at https://www.mdpi.com/article/10.3390/microorganisms13122854/s1, Figure S1. Multiplexing experimental set up and outcomes; Figure S2. Reproducibility of Positive controls; Figure S3. Target enrichment is agnostic to specimen matrix and NGS library preparation; Figure S4. Highly divergent virus coverage results from non-specific binding; Figure S5. Viruses detected in acute febrile illness samples from Asia; Table S1. The addition of an internal control does not affect viral detection; Table S2. teNGS and mNGS data for commercially sourced clinical specimens; Table S3. Serial dilution sequencing of viruses absent in CVRP probe set; Table S4. Divergent viruses with closer relatives in probe set are preferentially retained; Table S5. >65% identity is required for specific capture of divergent sequences; Table S6. ART statistical test of genome coverage by sequencing method; Table S7. GenBank and SRA data submission information.
