# Supplementary material for: teamNGS Balances Sensitivity for Viruses with Comprehensive Microbial Detection in Clinical Specimens
Source: Microorganisms. 2025 Dec 16;13(12):2854. doi: 10.3390/microorganisms13122854 (PMC12736096; doi:10.3390/microorganisms13122854)
Supplement: Supplementary file 1 [file microorganisms-13-02854-s001.zip › Supplemental Figures S1-S5.pdf]

NGS Run 1

8-plex	EMCV 10			
	EMCV 1			
	HIV 5000			
	HIV 1000			
	Zika 5000			
	Zika 1000			
	Covid 5000			
	Covid 1000			
32 -plex	EMCV 10	Neg 1	Neg 5	Neg 9
	EMCV 1	Neg 2	Neg 6	Neg 10
	HIV 5000	Neg 3	Neg 7	Neg 11
	HIV 1000	Neg 4	Neg 8	Neg 12
	Zika 5000	Low pos 1	Low pos 3	Low pos 5
	Zika 1000	Low pos 2	Low pos 4	Low pos 6
	Covid 5000	High pos 1	High pos 3	High pos 5
	Covid 1000	High pos 2	High pos 4	High pos 6

Plexity	Expected Virus	Sample	# Total Reads	Mapped Reads	% On-Target	% Genome Cov
32	HAV	High-1-2	386614	176282	45.6	100
32	HIV-1	High-2-2	737064	355276	48.2	95
32	Parvovirus	High-3-2	267754	52818	19.7	95
32	HAV	High-4-2	1314750	998643	76.0	100
32	HAV	High-5-2	1041914	799867	76.8	100
32	Parvovirus	High-6-2	2918966	1900737	65.1	98
32	HAV	Low-1-2	2983712	19231	0.6	100
32	HAV	Low-2-2	109766	16215	14.8	100
32	Dengue	Low-3-2	160170	0	0.0	
32	HBV	Low-4-2	191042	6692	3.5	98
32	Merkel Cell Polyomavirus	Low-5-2	203830	0	0.0	
32	HEV	Low-6-2	366902	0	0.0	
32	N/A	Negative-1-2	6088			
32	N/A	Negative-2-2	147812			
32	N/A	Negative-3-2	115746			
32	N/A	Negative-4-2	87580			
32	N/A	Negative-5-2	597142			
32	N/A	Negative-6-2	1289522			
32	N/A	Negative-7-2	1109118			
32	N/A	Negative-8-2	95238			
32	N/A	Negative-9-2	153630			
32	N/A	Negative-10-2	17802			
32	N/A	Negative-11-2	635176			
32	N/A	Negative-12-2	331658			

NGS Run 2

16 plex	EMCV 10	Neg 1	
	EMCV 1	Neg 2	
	HIV 5000	Neg 3	
	HIV 1000	Neg 4	
	Zika 5000	Low pos 1	
	Zika 1000	Low pos 2	
	Covid 5000	High pos 1	
	Covid 1000	High pos 2	
24-plex	EMCV 10	Neg 5	Neg 9
	EMCV 1	Neg 6	Neg 10
	HIV 5000	Neg 7	Neg 11
	HIV 1000	Neg 8	Neg 12
	Zika 5000	Low pos 3	Low pos 5
	Zika 1000	Low pos 4	Low pos 6
	Covid 5000	High pos 3	High pos 5
	Covid 1000	High pos 4	High pos 6

Plexity	Expected Virus	Sample	# Total Reads	Mapped Reads	% On-Target	% Genome Cov
16	HAV	High-1-2	644672	287742	44.6	100
16	HIV-1	High-2-2	1100950	479147	43.5	96
16	HAV	Low-1-2	4005700	26671	0.7	100
16	HAV	Low-2-2	157606	22643	14.4	100
16	N/A	Negative-1-2	9488			
16	N/A	Negative-2-2	249450			
16	N/A	Negative-3-2	222450			
16	N/A	Negative-4-2	158932			

Plexity	Expected Virus	Sample	# Total Reads	Mapped Reads	% On-Target	% Genome Cov
24	Parvovirus	High-3-2	268224	44920	16.7	94
24	HAV	High-4-2	751262	555463	73.9	100
24	HAV	High-5-2	1154430	860043	74.5	100
24	Parvovirus	High-6-2	3111088	1957125	62.9	98
24	Dengue	Low-3-2	205362	0	0.0	0
24	HBV	Low-4-2	258962	7795	3.0	98
24	Merkel Cell Polyomavirus	Low-5-2	242466	0	0.0	0
24	HEV	Low-6-2	518882	0	0.0	0
24	N/A	Negative-5-2	807174			
24	N/A	Negative-6-2	1426796			
24	N/A	Negative-7-2	1027412			
24	N/A	Negative-8-2				
24	N/A	Negative-9-2				
24	N/A	Negative-10-2	22702			
24	N/A	Negative-11-2	801692			
24	N/A	Negative-12-2	412352			

Supplemental Figure S1

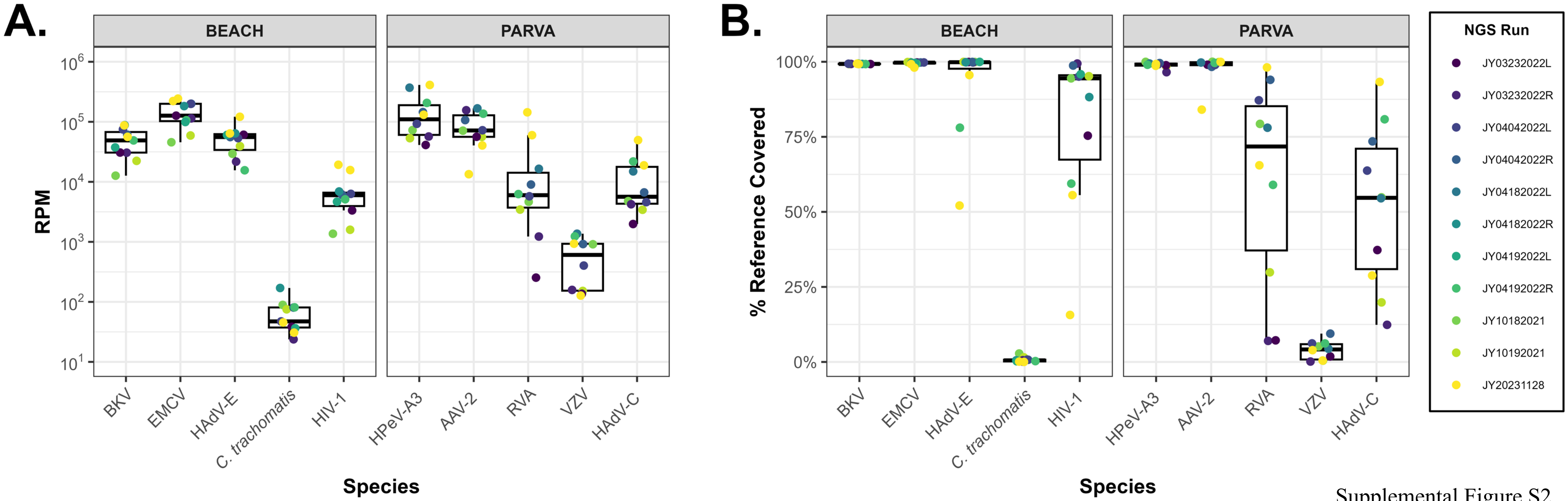

Supplemental Figure S2

A. Blood vs Plasma

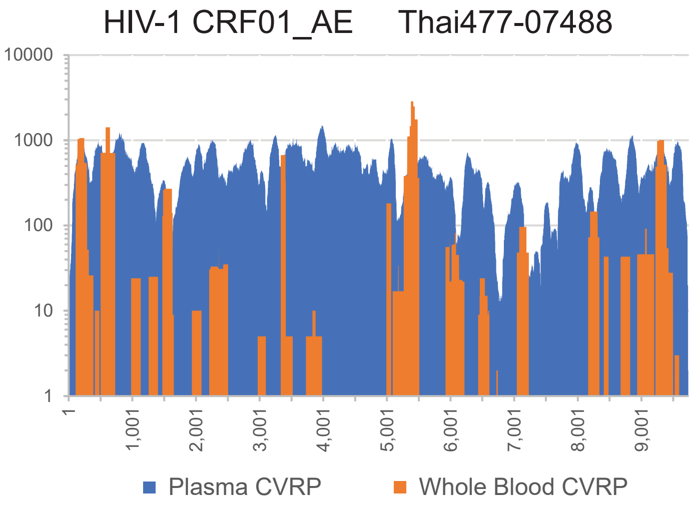

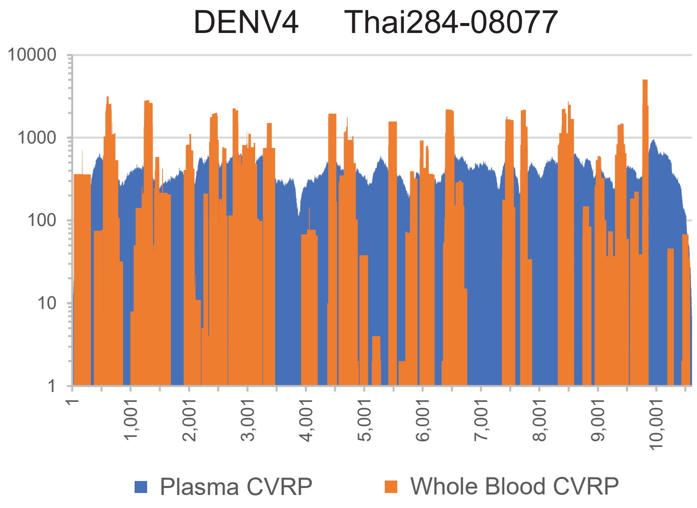

B. Nextera vs Quanta Bio

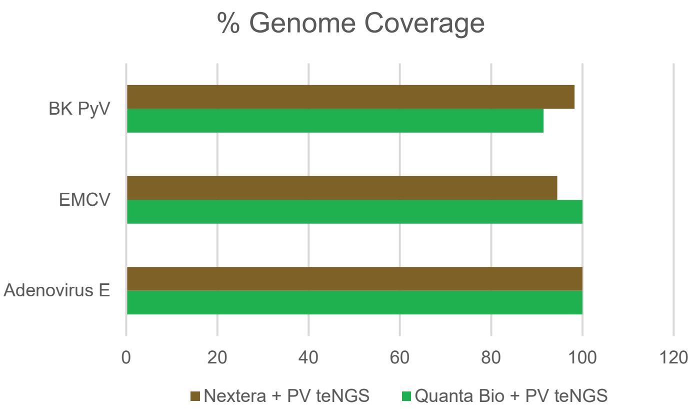

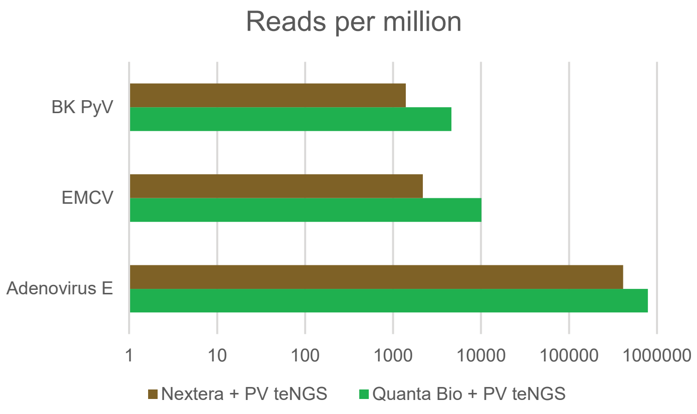

C. Quanta Bio mNGS vs teNGS

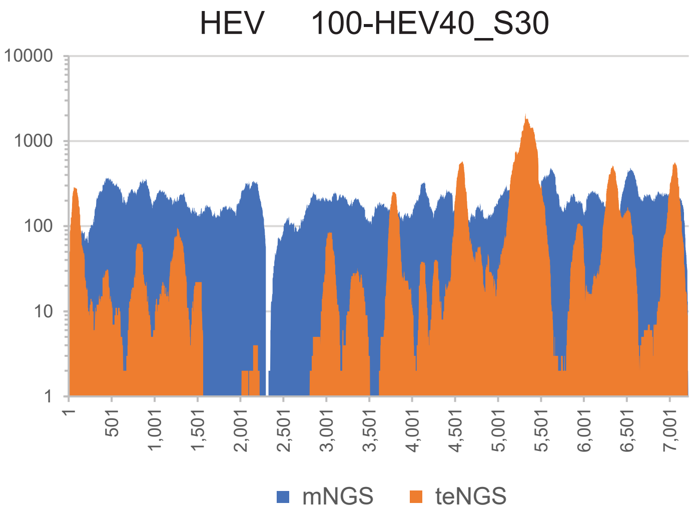

Supplemental Figure S3

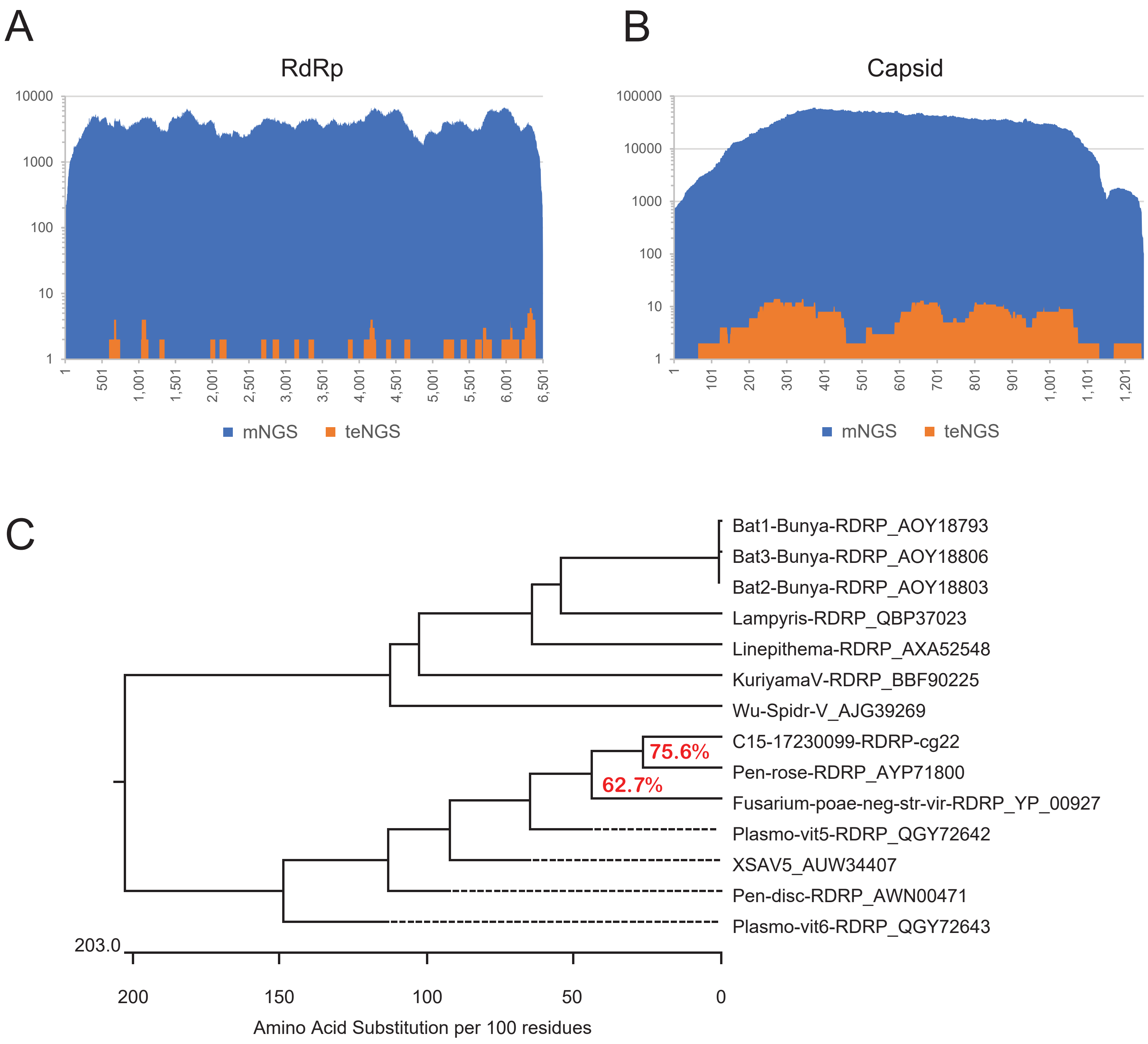

Supplemental Figure S4

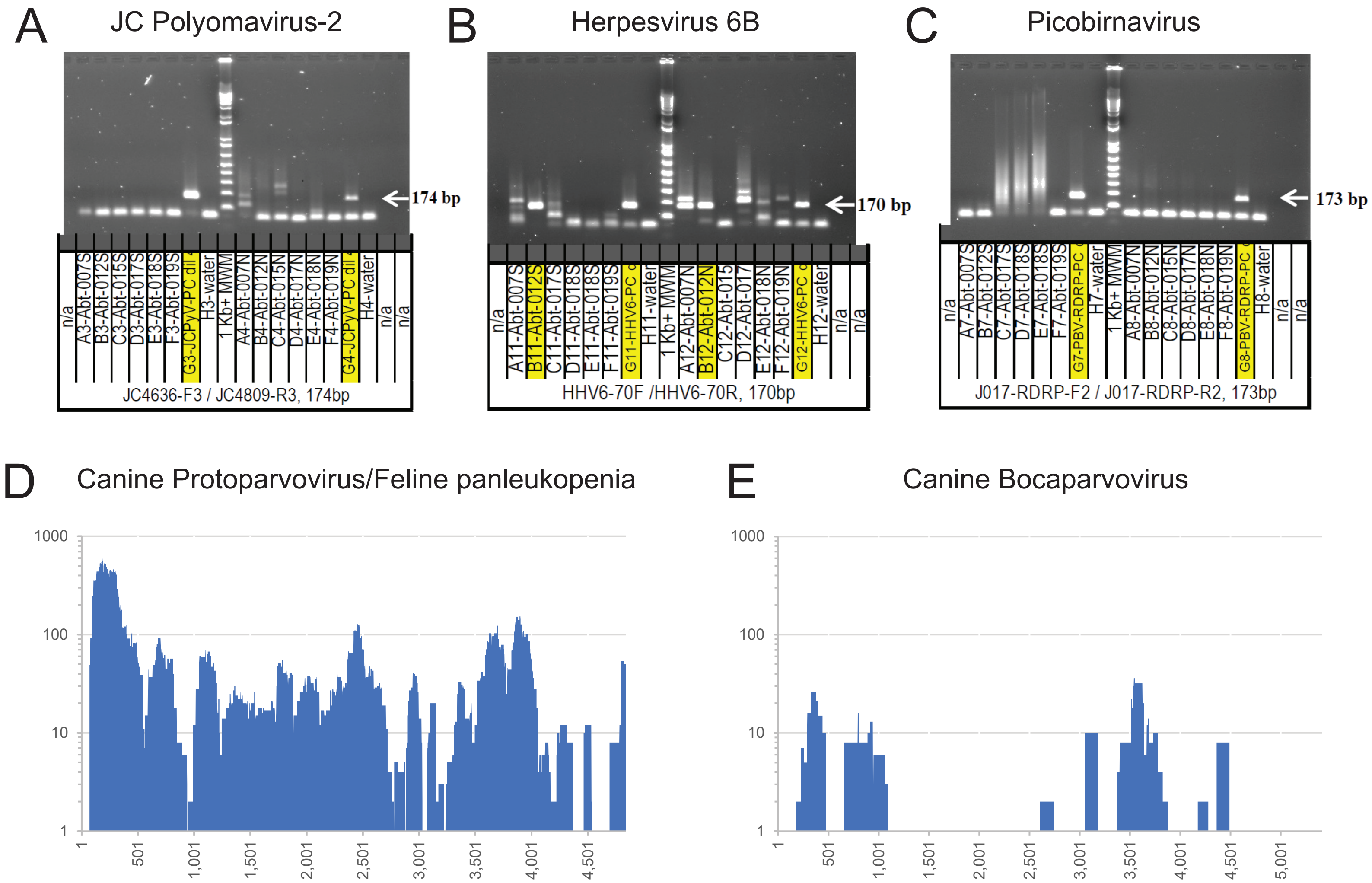

Supplemental Figure S5
