# Supplementary material for: teamNGS Balances Sensitivity for Viruses with Comprehensive Microbial Detection in Clinical Specimens
Source: Microorganisms. 2025 Dec 16;13(12):2854. doi: 10.3390/microorganisms13122854 (PMC12736096; doi:10.3390/microorganisms13122854)
Supplement: Supplementary file 1 [file microorganisms-13-02854-s001.zip › teamNGS Online Supplemental Information and Figures.pdf]

Online Supplementary Information for:

teamNGS balances sensitivity for viruses with comprehensive microbial detection in clinical specimens

Julie Yamaguchi[1,2,†], Gregory S. Orf[1,2,†], Jenna Malinauskas[1,2], Maximillian Mata[1,2], Sonja L. Weiss[1,2], Kenn Forberg[1,2], Todd V. Meyer[1,2], Peter O. Wiebe[1,2], Illya Mowerman[1,2], Stanley J. Piotrowski[1,2], Daniel Glownia[1,2], Mary A. Rodgers[1,2], John Hackett Jr[1], Yupin Suputtamongkol[2,3], Pakpoom Phoompoung[2,3], Selvamurthi Gomathi[2,4], Amrose Pradeep[2,4], Sunil S. Solomon[2,4,5], Nicholas Bbosa[2,6,7], Pontiano Kaleebu[2,6,7], Ambroise D. Ahouidi[2,8], Souleymane Mboup[2,8], Austin F. Sequeira[9], Arinobu Tojo[10], Gavin A. Cloherty[1,2], Michael G. Berg[1,2]

† co-first authors
[1] Infectious Disease Research, Abbott Laboratories, Abbott Park, IL USA
[2] Abbott Pandemic Defense Coalition
[3] Faculty of Medicine, Siriraj Hospital Mahidol University, Bangkok, Thailand
[4] YRG Care, Chennai, India
[5] Johns Hopkins University, School of Medicine, Baltimore, MD USA
[6] Uganda Virus Research Institute, Kampala, Uganda
[7] MRC/UVRI & LSHTM Uganda Research Unit
[8] Institut de Recherche en Santé, de Surveillance Épidémiologique et de Formations (IRESSEF), Dakar, Senegal
[9] SlieaGen, LLC, Austin, TX USA
[10] Institute of Medical Science, University of Tokyo, Japan (current address: Tokyo Medical and Dental University)
Corresponding author: michael.berg@abbott.com

Supplementary Methods

Metagenomic Library preparation. Serum or plasma were treated with Benzonase (MilliporeSigma, Burlington, MA, USA) to deplete host DNA, then nucleic acids were extracted on an Abbott *m*2000 platform or KingFisher Apex using the MagMax Viral/Pathogen Nucleic Acid Isolation kit (ThermoFisher Scientific, Waltham, MA, USA). Metagenomic cDNA libraries were synthesized from total nucleic acids on an epMotion liquid handler (Eppendorf North America, Enfield, CT, USA) using SuperScriptIV and Sequenase v2.0 (ThermoFisher Scientific, Waltham, MA, USA) or manually using qScript XLT cDNA SuperMix (QuantaBio, Beverly, MA, USA) and Sequenase v2.0. cDNAs were barcoded for NGS on an epMotion liquid handler using an Illumina Nextera XT v2 library prep kit (Illumina, Inc, San Diego, CA, USA) and custom IDT-Nextera adapters (Integrated DNA Technologies, Coralville, IA, USA), or manually using a sparQ DNA Frag and Library Prep kit and UDI adapters (QuantaBio).

CVRP Library Preparation. Sets of 24 mNGS libraries were pooled for enrichment of viral sequences using the Comprehensive Viral Research Panel (CVRP, Twist Biosciences, South San Francisco, CA, USA), composed of over 1MM unique probes covering reference sequences for 3,153 viruses, including 15,488 different strains, as previously described (Berg et al. 2020; Orf et al. 2024) and according to manufacturer's protocol. CVRP target enriched (CVRPte) libraries were sequenced on Illumina MiSeq or NextSeq 1000 instruments. NGS fastq files were processed to remove low quality and human sequences and to identify remaining microbial reads using DiVir 3.0, an in-house metagenomics and virus discovery pipeline.

CVRP wash stringency. To compare two different wash conditions, the hybridization reaction was doubled to accommodate splitting the hybridized pool into two different pools- each to receive either the stringent or lenient wash condition. Replicate pools were made to evaluate the two wash conditions: Lenient wash and Stringent wash. The Lenient wash condition consisted of the following: (1) room temperature wash buffer 1, (1) room temperature wash buffer 2 wash. The Stringent wash buffer condition consisted of the following washes: (1) room temperature wash buffer 1, (3) 48C wash buffer 2 washes with 48C 5 min incubation after each addition of wash buffer 2. The pools were amplified for 20 cycles and cleaned up application of Twist purification beads. Twenty-eight samples were sequenced on the Illumina MiSeq – the Lenient wash condition pool ~~on one run~~ and the Stringent wash condition pool were loaded on separate MiSeq runs.

teamNGS Library Preparation. To test the teamNGS concept, we varied two dependent variables: 1) the ratio of metagenomic to target-enriched libraries and 2) the allotted read capacity per library. In our laboratory, metagenomic NGS libraries are generally constructed in a 96-well format. The plate is then split into 4 groups, which are each pooled together (*i.e.*, libraries 1-24 are pool 1, libraries 25-48 are pool 2, etc.) for viral enrichment by CVRP. To construct teamNGS, after CVRP enrichment, we made corresponding equimolar metagenomic library pools with the same identities as their CVRP counterparts (*i.e.*, again, libraries 1-24 are pool 1, libraries 25-48 are pool 2, etc., each first diluted to 2 nM). Each metagenomic and CVRP pool were then diluted (or confirmed) to be at a 2-nM working concentration. Each teamNGS run on a NextSeq P2 kit consisted of 48 libraries, so two pools were sequenced together: to test a 10% ratio, 1 µl of each CVRP pool (*e.g.*, pools 1 and 2, corresponding to libraries 1-24 and 25-48, respectively) and 9 µl of each metagenomic pool (*e.g.*, pools 1 and 2, corresponding to libraries 1-24

and 25-48, respectively) were mixed together to produce a 20-µl sequencing pool at 2 nM *total DNA concentration*. To test a 20% ratio, the volumes were correspondingly changed to 2 µl of each CVRP pool and 8 µl of each metagenomic pool. (see **Main Text, Figure 6A** for a schematic)

When sequencing Nextera-XT libraries on the NextSeq 1000, we have empirically determined a loading concentration of 650 pM to be ideal; thus, we mix 7.89 µl of the 2-nM teamNGS sequencing pool with 16.11 µl of Illumina's RSB+Tween-20 and 1 µl of 1-nM PhiX control. Twenty microliters of this final solution are added to a NextSeq P2 kit, which allots for roughly 8.33 MM paired-end reads to be collected for each of the 48 libraries.

For testing the second dependent variable (allotting read capacity), we utilized a NextSeq P1 kit (4 times less capacity than a P2 kit), while also limiting the sequencing run to 24 libraries (*i.e.*, 1 pool, thus half of the number of libraries as before). To test the 10% teamNGS ratio, 2 µl of the CVRP pool (*e.g.*, pools 1, corresponding to libraries 1-24) and 18 µl of the metagenomic pool (*e.g.*, pool 1, corresponding to libraries 1-24) were mixed to produce a 20-µl sequencing pool at 2 nM *total DNA concentration*. The same final dilution and load scheme as above was used, which allowed for the collection of roughly 4.16 MM paired-end reads per library.

PCR primers for Japan samples Primers for traditional PCR confirmation of viruses in the Japan samples were designed using PrimerSelect (DNASTAR, Inc, Madison, WI). Two sets of oligos were designed for each virus, to allow for dual detection.

Human herpesvirus 6, oligo set 1:
HHV6-25F, ACTCCATGTCTCGTCAAAGCC and HHV6-25R, TTGCGTCGTTCATCATTTCCG;
Human herpesvirus 6, oligo set 2:
HHV6-70F, TGGTTGCTGAATTGCTTTCCG and HHV6-70R, CAAGCCATGTCCATCGTTGTG;
Human picobirnavirus, oligo set 1:
J017-RDRP-F1, GGCAAGTTCAAAGTATGCAGGG and J017-RDRP-R1 CTCACGGAGGGGATGGTACTTA;
Human picobirnavirus, oligo set 2:
J017-RDRP-F2, GAAGGCTGTAGACCTAGCGAAG and J017-RDRP-R2, CCCAGCCTGTATTTATCCCCTT;
JC Polyomavirus, oligo set 1:
JC732-F1, TGGAGAGGCTGCTGCTACTA and JC889-R1, ATGCAGCAAACCCAGCTACA;
JC Polyomavirus, oligo set 2:
JC4636-F3, GTCTCCAAGAACCTTCTCCCAG and JC4809-R3, GTGCATTGCCCCTGTTTAATGT.

The PCR reactions were run using the Applied Biosystems AmpliTaq DNA Polymerase kit (ThermoFisher Scientific, Waltham, MA) according to manufacturer's instructions, using metagenomic NGS libraries as the template. The thermocycle was 98°C for 1 min, then 55 cycles of the following: 94°C for 15 sec, 50°C for 30 sec, and 72°C for 30 sec.

Supplementary Figure Legends

Figure S1. **Multiplexing experimental set up and outcomes** Multiplexing effects on hybridization captures and sequencing was tested in two runs, each with 40 specimens. In NGS Run 1 (top), the two dilutions of EMCV, HIV, Zika, and SARS-CoV-2 contrived samples shown in Figures 2B & 2C were either captured alone (8-plex) or in the presence of 12 virus-negative, 6 low titer virus-positives, and 6 high titer virus positives (32-plex). Note that model virus libraries were tagged with different barcodes, notated in red or orange, respectively. Mapping results are shown for the added low and high titers viruses included within the 32-plex capture. In NGS Run 2 (bottom), the two dilutions of EMCV, HIV, Zika, and SARS-CoV-2 contrived samples shown in Figures 2B & 2C were either captured in the presence of 4 virus-negative, 2 low titer virus-positives, and 2 high titer virus positives (16-plex) or in the presence of 8 virus-negative, 4 low titer virus-positives, and 4 high titer virus positives (24-plex). Once again, each set of model viruses had distinct barcodes and mapping metrics for the additional viruses are reported. Note that just as for model viruses in Figures 2B & 2C, detection of other viruses derived from clinical specimens was unaffected by the degree of multiplexing.

Figure S2. Reproducibility of Positive controls. Within each capture of 24 libraries, a positive control is included consisting of a cocktail of viruses (and bacteria) each diluted to log 3.0 cp/ml in normal human plasma. The BEACH control consists of BK polyomavirus, EMCV, human Adenovirus E, Chlamydia trachomatis, and HIV-1. The PARVA control consists of Parechovirus A3, Adeno-associated virus-2, Rotavirus A, Varicella Zoster Virus, and human Adenovirus C. Recovery of each virus varied relative to each other but was consistent from one experiment to the next. **A**) Reads per million results for each species are reported for eleven independent experiments. **B)** Percent genome coverage for each species reported for ten independent experiments.

Figure S3. Target enrichment is agnostic to specimen matrix and NGS library preparation. A) teNGS libraries (derived from Nextera XT libraries) were prepared from the same individual using plasma (blue) and whole blood (orange). Examples are shown for HIV-1 CRF01_AE (left) and DENV4 (right) strains. **B)** Pan-viral (PV) teNGS libraries for a positive control containing BK polyomavirus, EMCV, and adenovirus E were prepared from Superscript | Nextera libraries (brown) and XLT | QuantaBio libraries (green). Percent genome coverage (left) and reads per million (right) are reported for each virus. **C)** mNGS (color) and teNGS (color) libraries were prepared using the Quanta Bio cDNA synthesis and NGS library prep reagents. An example is shown for Hepatitis E virus (HEV).

Figure S4. Highly divergent virus coverage results from non-specific binding. A novel bunyavirus related to Fusarium fungal (Ixodes) bunyavirus was detected in abundance in the mNGS library of Cerba sample C15-17230099. Coverage plots derived from mNGS (blue) and teNGS (orange) libraries are shown for **A)** the RdRp (L; left) and **B**) capsid (S; right) encoding segments. The paucity of teNGS reads indicate they were not selectively enriched. **C**) Maximum-likelihood tree of RdRp amino acid sequences for the novel virus and other related fungal and invertebrate bunyaviruses. The novel virus lacks an M segment and its RDRP bears only 75.6% AA identity to Penicillium roseopurpureum negative ssRNA virus 1 and 62.7% identity to Fusarium poae negative strand virus.

Figure S5. Viruses detected in acute febrile illness samples from Asia. A panel of n=7 samples from Japan with AFI following hematopoietic stem cell transplant were evaluated by both mNGS and teNGS

and confirmation was attempted by PCR: **A**) JC PyV-2, **B**) HHV-6B, **C**) PBV. Two different library preparations were used at template: NuGen SPIA (left) and Illumina Nextera XT (right). Positive controls (lane 7) or positive samples are highlighted in yellow. AFI samples (n=150) from India were evaluated only by teNGS. **D**) Genome coverage plot of a Canine protoparvovirus / feline panleukopenia virus found in sample APDC066. **E**) Genome coverage plot of a Canine bocaparvovirus found in sample APDC076.

Supplementary Table Legends

Table S1. The addition of an internal control does not affect viral detection. EMCV virus was selected as an internal control and added to samples during the lysis step of extraction. Table rows alternate between samples with ('S') or without IC spiked-in. Mapped reads are reported for all model viruses (present or not) to assess the level of index-hopping. The detection of these viruses was not affected by the presence of EMCV.

Table S2. teNGS and mNGS data for commercially sourced clinical specimens. Samples with confirmed or suspected diagnosis, as well as those considered 'discovery' without a diagnosis and exhibiting acute febrile illness symptoms, were evaluated by mNGS and teNGS. The first three columns list the vendor source and their PCR and serology data. NGS library preparation types are either Nextera or QuantaBio, and either unenriched mNGS or target enriched by Pan-viral (PV; an earlier 'beta' version of CVRP) or CVRP. The virus detected by NGS is listed, along with the indicated mapping metrics.

Table S3. Serial dilution sequencing of viruses absent in CVRP probe set. Ten-fold serial dilutions of Mount Elgon bat, Nodamura, Simian Hemorrhagic Fever, and Wanowrie viruses were spiked into healthy donor plasma, mNGS and teNGS libraries were prepared, and each was sequenced. Mapping results are reported for the 10^{-2} and 10^{-4} dilutions for mNGS and for the 10^{-2}-10^{-5} dilutions for teNGS. Note that Nodamura virus has two segments and Wanowrie has three segments (L, M, S).

Table S4. Divergent viruses with closer relatives in probe set are preferentially retained. Mapping results for 10^{-4} dilutions of mNGS and teNGS libraries for Mount Elgon bat, Nodamura, Simian Hemorrhagic Fever, and Wanowrie viruses. Partial genome coverage of teNGS compared to mNGS at this dilution suggested some viral sequences might be retained through specific interactions with probes compared to those that were not. The percentage of human, bacterial, and viral reads for each species were tracked with each library preparation. The fold reduction column indicates the decrease in percentage of viral reads going from mNGS to teNGS.

Table S5. >65% identity is required for specific capture of divergent sequences. Alignment intervals of 1000 nucleotides or greater for Wanowrie virus segments and Mt Elgon Bat virus were evaluated against related viruses. Percent identity was reported for the closest relative as well as the average identity of species in the genus within this interval. Alignment coordinates where strong selective enrichment was observed at the 10^{-4} dilution were highlighted.

Table S6. ART statistical test of genome coverage by sequencing method. An Aligned Rank Transform test of samples in Figures 6B & 6C was performed to compare genome coverage across sequencing methods. Estimate values indicate the difference in coverage for methods for a given comparison and are either positive (A>B) or negative (A<B). P-values < 0.05 are considered significant.

Table S7. GenBank and SRA data submission information. GenBank, Bioproject, Biosample, and Sequence Read Archive accessions are reported for patient sequences submitted to public repositories. Genomes with >90% coverage were included.
